# Conservative management versus invasive management of significant traumatic pneumothoraces in the emergency department (the CoMiTED trial): a study protocol for a randomised non-inferiority trial

**DOI:** 10.1136/bmjopen-2024-087464

**Published:** 2024-06-17

**Authors:** Nicola M Blythe, Katherine Coates, Jonathan R Benger, Ammar Annaw, Jonathan Banks, Clare Clement, Madeleine Clout, Antoinette Edwards, Daisy Gaunt, Rebecca Kandiyali, J Athene Lane, Fiona Lecky, Nick A Maskell, Chris Metcalfe, Marie Platt, Sophie Rees, Jodi Taylor, Julian Thompson, Steven Walker, Douglas West, Edward Carlton

**Affiliations:** 1 Bristol Trials Centre, University of Bristol, Bristol, UK; 2 North Bristol NHS Trust, Bristol, UK; 3 University Hospitals Bristol and Weston NHS Foundation Trust, Bristol, UK; 4 University of the West of England, Bristol, UK; 5 The University of Manchester, Manchester, UK; 6 University of Warwick, Coventry, UK; 7 The University of Sheffield, Sheffield, UK; 8 Salford Royal NHS Trust, Salford, UK; 9 University of Bristol, Bristol, UK

**Keywords:** accident and emergency medicine, Randomized Controlled Trial, trauma management, traumatic pneumothorax, conservative management

## Abstract

**Introduction:**

Traumatic pneumothoraces are present in one of five victims of severe trauma. Current guidelines advise chest drain insertion for most traumatic pneumothoraces, although very small pneumothoraces can be managed with observation at the treating clinician’s discretion. There remains a large proportion of patients in whom there is clinical uncertainty as to whether an immediate chest drain is required, with no robust evidence to inform practice. Chest drains carry a high risk of complications such as bleeding and infection. The default to invasive treatment may be causing potentially avoidable pain, distress and complications. We are evaluating the clinical and cost-effectiveness of an initial conservative approach to the management of patients with traumatic pneumothoraces.

**Methods and analysis:**

The CoMiTED (Conservative Management in Traumatic Pneumothoraces in the Emergency Department) trial is a multicentre, pragmatic parallel group, individually randomised controlled non-inferiority trial to establish whether initial conservative management of significant traumatic pneumothoraces is non-inferior to invasive management in terms of subsequent emergency pleural interventions, complications, pain, breathlessness and quality of life. We aim to recruit 750 patients from at least 40 UK National Health Service hospitals. Patients allocated to the control (invasive management) group will have a chest drain inserted in the emergency department. For those in the intervention (initial conservative management) group, the treating clinician will be advised to manage the participant without chest drain insertion and undertake observation. The primary outcome is a binary measure of the need for one or more subsequent emergency pleural interventions within 30 days of randomisation. Secondary outcomes include complications, cost-effectiveness, patient-reported quality of life and patient and clinician views of the two treatment options; participants are followed up for 6 months.

**Ethics and dissemination:**

This trial received approval from the Wales Research Ethics Committee 4 (reference: 22/WA/0118) and the Health Research Authority. Results will be submitted for publication in a peer-reviewed journal.

**Trial registration number:**

ISRCTN35574247.

**STRENGTHS AND LIMITATIONS OF THIS STUDY**

- This is a pragmatic trial; once the initial decision has been made and patients have been allocated a treatment arm, all subsequent care and interventions are at the discretion of treating clinical teams.
- Patients will be recruited from the whole of the trauma spectrum to ensure results can be generalisable across the diverse trauma population.
- The trial involves economic evaluation to determine the clinical and cost-effectiveness of initial conservative management versus invasive management of traumatic pneumothoraces.
- The trial has an integrated qualitative study in order to assess the acceptability of initial conservative management to patients and clinicians.
- Blinding to treatment allocation is not possible for clinicians or participants; only clinicians adjudicating primary outcome and researchers evaluating outcomes for the analyses will be blinded to treatment group.

## Introduction

Injury is a leading cause of death among adults aged <45 years.^1^ Traumatic pneumothoraces are present in one of five victims of severe trauma.^2 3^ We estimate from prior observational and survey work^4 5^ that around half of patients admitted to hospital with traumatic pneumothoraces will be treated with the insertion of a chest drain. Current guidelines advise chest drain insertion for most traumatic pneumothoraces, although very small pneumothoraces can be managed with observation at the treating clinician’s discretion.^6 7^ For some patients with very large pneumothoraces, chest drain placement can reduce the risk of cardiorespiratory compromise.^8^ However, there remains a large proportion of patients in whom there is clinical uncertainty as to whether an immediate chest drain is required.^4^ Chest drains carry a high risk of complications, such as bleeding and infection, in 15–30% of patients.^9^ There is a lack of robust evidence to inform practice, and the default to invasive treatment may cause potentially avoidable patient harm.

In an analysis of >600 patients with traumatic pneumothoraces from 2012 to 2016, obtained from Trauma Audit & Research Network (TARN) data, 90% of patients treated without a chest drain did not require subsequent intervention,^5^ suggesting a potential role for conservative management. However, in this analysis, 50% of patients were initially treated with a chest drain and there was considerable clinical variation in those selected for this invasive procedure. In a 2020 international survey of 222 emergency physicians,^4^ using clinical vignettes of larger traumatic pneumothoraces, over 60% of clinicians would elect to insert a chest drain in the emergency department (ED), even without clinical compromise. Therefore, based on the observational studies and lack of robust data, we designed a randomised controlled trial (RCT) to assess the clinical and cost-effectiveness of an initial conservative approach to the management of patients with traumatic pneumothoraces. If we demonstrate that this approach achieves similar clinical outcomes, it will reduce the use of a painful, invasive and potentially harmful management strategy.

Prior to the start of the trial, we searched Medline for systematic reviews, and Medline, Embase, Cochrane Central, ClincalTrials.gov and the World Health Organisation (WHO) trials registry for trials published. One systematic review from 2010 evaluated three small (total n=101) RCTs examining the safety of conservative management in small traumatic pneumothoraces.^8^ This review suggested that conservative management may be at least as safe and effective as chest drain insertion. A further multicentre RCT of small pneumothoraces in severely injured patients in Canada concluded in 2021.^10^ These patients (142 in total) were all receiving positive pressure ventilation and current guidelines suggest chest drain insertion in all patients undergoing ventilation.^2 4^ The results showed no difference in mortality or intensive care unit (ICU) or hospital length of stay between patients who were conservatively managed and those who had chest drains inserted. The authors concluded that small traumatic pneumothoraces may be safely observed in patients undergoing ventilation and that the complications of chest drains remain unacceptably high. By including only patients undergoing ventilation (which is around 30% of the traumatic pneumothorax population in the UK^5^), the Canadian study did not fully address conservative management in the broader trauma population, as we are in this trial.

## Aims and objectives

The Conservative Management in Traumatic Pneumothoraces in the Emergency Department (CoMiTED) trial will test whether initial conservative management of significant traumatic pneumothoraces is non-inferior to invasive management in terms of subsequent emergency pleural interventions, complications, pain, breathlessness and quality of life.

Specific objectives are:

- To establish if initial conservative management is non-inferior to invasive management regarding subsequent emergency pleural intervention over 30 days (or until death if sooner).
- To determine whether conservative management improves health-related quality of life and other patient-reported outcomes.
- To determine the clinical and cost-effectiveness of initial conservative management versus invasive management of traumatic pneumothoraces by measuring resource use, mortality and costs over the 6 months following injury.
- To assess acceptability of initial conservative management to patients and clinicians.

## Methods and analysis

### Trial design

The CoMiTED trial is a pragmatic multicentre, parallel group, individually randomised controlled non-inferiority trial with an economic evaluation and integrated qualitative study.

### Setting

The trial will recruit patients from approximately 40 National Health Service (NHS) major trauma centres and trauma units across the UK.

### Trial population

Inclusion and exclusion criteria are detailed in table 1.

**Table 1:** Inclusion and exclusion criteria

Inclusion criteria	Exclusion criteria
Presenting with traumatic pneumothorax/pneumothoraces	Treating clinician(s) believes injuries are incompatible with life
(Believed to be) 16 years and over	Patient in respiratory arrest
Treating clinician(s) believes either a chest drain or conservative management is a suitable initial treatment option	Haemothorax (associated with pneumothorax) requiring a chest drain in the opinion of the treating clinician(s)*
	Clinical or imaging evidence of tension pneumothorax in either lung at the point of randomisation
	Prisoner

Special circumstances: in patients presenting with bilateral chest injury, if one lung of the patient qualifies, the patient can be enrolled, providing no exclusion criteria are met. Treatment of the eligible side follows the randomisation assignment, with the other side treated according to usual practice. If both sides qualify, both sides receive treatment according to the randomisation assignment. Patients who have received prehospital thoracostomies may still be enrolled, provided they fulfil the eligibility criteria. Where a participant who has received a prehospital thoracostomy is randomised to conservative management, local practice should be followed.

*Patients with an associated haemothorax are excluded due to this being a predictor of failure of conservative management.^5^

### Primary outcome

The primary outcome is a binary measure of the need for one or more subsequent emergency pleural interventions in the eligible lung(s), from the point of randomisation up to 30 days. This excludes chest drain insertion in the ED for those allocated to the chest drain (control) group.

Reasons for subsequent emergency chest drain insertion may include (but are not limited to): clinically significant symptoms persisting despite adequate analgesia; chest pain or breathlessness preventing activity; a patient is unwilling to continue with conservative treatment; the patient’s condition becomes physiologically unstable presumed secondary to pneumothorax; repeat chest radiograph shows an enlarging pneumothorax with physiological instability. Reasons for subsequent emergency pleural intervention are determined by local practice and recorded but are not controlled.

Whether a subsequent pleural intervention is deemed to be an emergency is adjudicated by a panel made up of independent expert clinicians from relevant specialties. The clinicians are blinded to allocation and presented with clinical and imaging vignettes of what happened to each participant and subsequently asked to determine whether, in their opinion, any subsequent pleural intervention that occurred within 30 days of randomisation was required due to an emergency. Consensus agreement is obtained by two members of the panel.

### Secondary outcomes

The secondary outcomes will capture any differences between the allocated groups in terms of reduced pain, complications and improved health-related quality of life in the short to medium term, as well as inform a formal cost-effectiveness analysis.

Secondary outcomes are as follows: (1) all pleural interventions (including chest drain insertion in the ED) up to 30 days; (2) all complications of pleural intervention up to 30 days; (3) total days of pleural drainage up to 30 days; (4) patient-reported pain,^11^ function and breathlessness^12^ at baseline, 30 days, 3 and 6 months; (5) quality of life^13 14^ at baseline, 30 days, 3 and 6 months; (6) total length of stay (hospital, critical care (including high-dependency unit) admission and readmission) up to 30 days; (7) adjudicated mortality at 30 days (pneumothorax or chest injury related); (8) all-cause mortality at 6 months; (9) cost per quality-adjusted life year (QALY) at 6 months; (10) patient views and experiences of conservative management/chest drain; and (11) clinician views of conservative management/chest drain. For this trial, baseline patient-reported outcome measures (PROMs) can be collected from as soon as feasible following randomisation and after treatment delivery (where appropriate) up to 7 days post-randomisation.

### Sample size

We aim to recruit 750 participants and conduct approximately 25 patient interviews for the integrated qualitative study over 37 months (August 2022–September 2025).

Observational data suggest that 10% of our trial population will require emergency pleural intervention following conservative management.^5^ Our group recently identified a reintervention rate of 10% following initial chest drain insertion in a single UK site^5^ and therefore anticipate the incidence of the primary outcome in the control group to be at least 10%.

Our patient and public involvement (PPI) contributors unanimously support the potential advantages of initial conservative management, such as avoiding an invasive procedure, improved mobilisation after injury and reduced longer-term pain. However, they also recognise the need to balance these benefits against the risk of avoidable harm. When asked, our PPI group felt that an increase of 5–10% in subsequent emergency pleural intervention would be acceptable compared with usual care, given the anticipated reduction in the overall number of chest drains in the intervention group. These views have been used to select a non-inferiority margin of 7.5%. We will conclude that the trial population can be safely managed conservatively if the incidence of subsequent emergency pleural intervention is no more than 7.5% higher in the intervention group than in the control group. If the incidence of the primary outcome is 10% in both groups, a sample size of 674 (337 in each group) will allow non-inferiority to be concluded with 90% power, when comparing a one-sided 97.5% confidence interval (CI), for the absolute difference in primary outcome incidence, to a non-inferiority margin of 7.5%. Allowing 10% loss to follow-up increases the total sample size to 750.

### Patient approach, recruitment and randomisation

**Figure 1:**
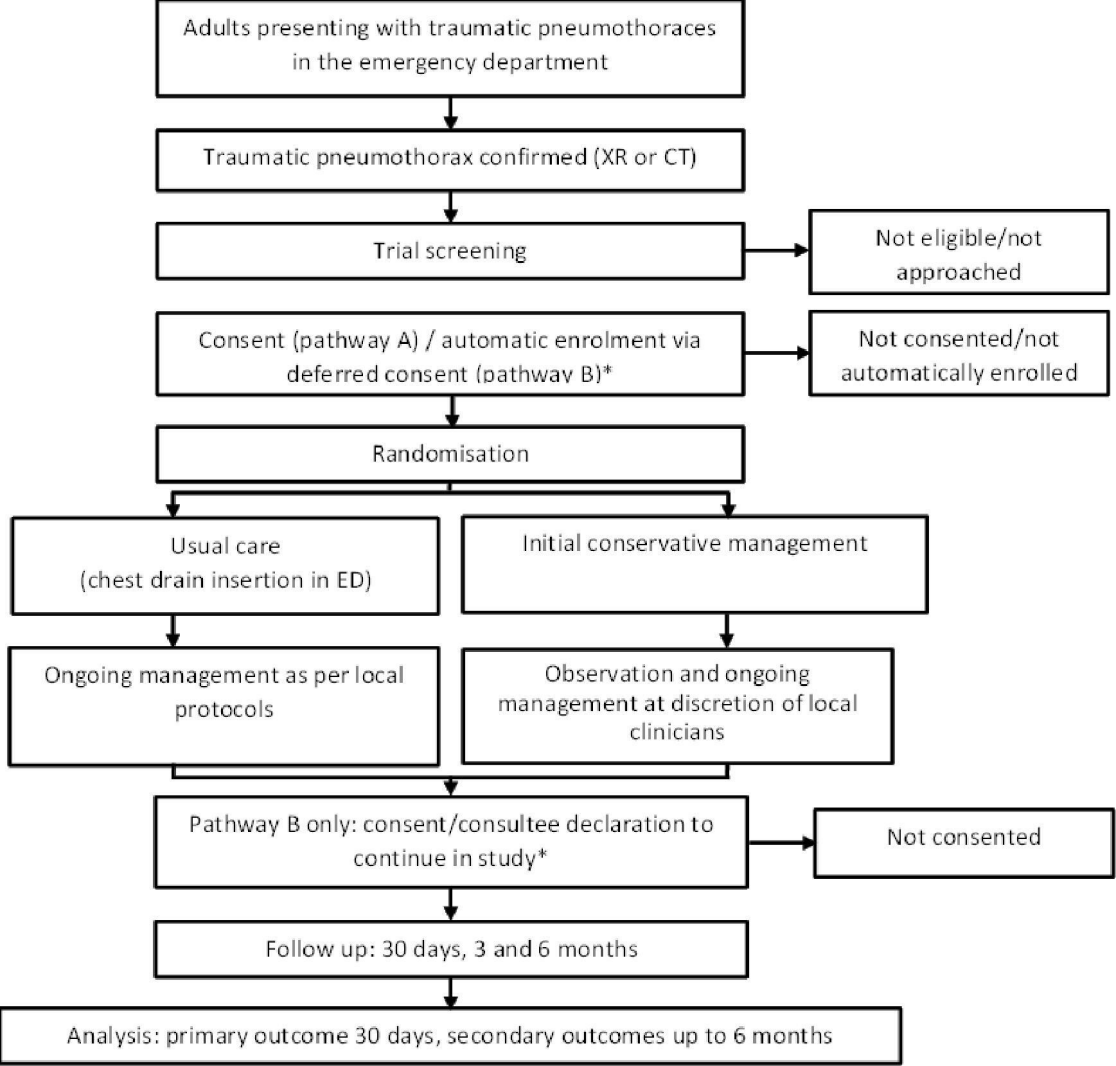
Trial schema illustrating the pathway for CoMiTED participants. *It should be noted that patients without capacity are not being recruited in Scotland (Pathway B in England, Wales and Northern Ireland only). ED, emergency department; XR, X-ray.

Following eligibility assessment, eligible patients undergo a capacity assessment. If the patient has capacity, they are approached in the ED for their consent to take part. Where patients are judged to be unable to provide informed consent for themselves, then patients can be automatically enrolled under the waiver of consent (in countries where the waiver of consent is permitted for non-Clinical Trials of Investigational Medicinal Products). If patients regain capacity within 7 days post-randomisation, they are approached and asked whether they wish to provide consent to continue in the trial. If patients do not regain capacity within 7 days of randomisation, a member of the research team seeks advice from a personal consultee or, if unavailable, a nominated consultee. The participant pathway is shown in figure 1.

Patients are randomised in the ED immediately after traumatic pneumothorax has been diagnosed and consent provided/waiver of consent applied. Participants are allocated in a 1:1 ratio to either ‘chest drain insertion in the ED (control group)’ or ‘initial conservative management (intervention group)’ using a secure web-based randomisation system (Sealed Envelope, https://www.sealedenvelope.com/). Randomised allocation is minimised by three factors: ‘trial site’, ‘currently ventilated’ and ‘penetrating injury’.

### Trial intervention

In the intervention (initial conservative management) group, the treating clinician is advised to manage the participant without chest drain insertion and undertake observation and admission to a hospital ward or ICU. Given the pragmatic nature of this trial, all subsequent interventions and further imaging to evaluate pneumothorax resolution after the point of randomisation are at the discretion of the treating clinical teams.

In the control group (chest drain insertion in the ED), the treating clinician is advised to insert a chest drain. Specific details of the procedure (including anaesthesia and insertion technique) are at the discretion of treating clinicians but will be recorded for trial purposes.

### Data collection

Trial data are collected at baseline, 30 days, 3 months and 6 months and recorded by participating site team members onto case report forms (CRFs) and participant-completed questionnaires. Table 2 depicts the key assessments/outcome measures and participant-related procedures scheduled at various trial time points. These are entered into a REDCap database^15^ for data cleaning and analysis. Access to the database is via a secure password-protected web interface.

**Table 2:** Schedule of essential data capture and participant-related procedures

Data collection time point (→)	In the emergency department (ED)		Post-randomisation follow-ups		
Data capture/key trial procedure (↓)	Recruitment (day 0)	Post-recruitment (baseline)	30 days	3 months	6 months
Screening, consent & randomisation	X				
Case report form including safety reporting	X	X	X	X	X
Sociodemographic details		X			
Injury details		X			
Injury Severity Score		X			
Comorbidities		X			
National Early Warning Score (routinely collected)		X			
PROMs; pain (Brief Pain Inventory)		X	X	X	X
PROMs; function (Brief Pain Inventory)		X	X	X	X
PROMs; breathlessness (MRC Dyspnoea Scale)		X	X	X	X
PROMs; quality of life (EQ-5D-5L)		X	X	X	X
PROMs; Impact Events Scale			X	X	X
Patient-completed resource use questionnaire				X	X
Pleural interventions			X		
Complications			X		
Days of pleural drainage			X		
Length of stay (hospital and critical care (including HDU) admission and readmission)			X		
Details of reattendances to A&E or unplanned readmissions within 30 days			X		
Mortality			X		X
Qualitative interviews			X		X

A&E, accident and emergency; HDU, high-dependency unit; MRC, Medical Research Council; PROMs, patient-reported outcome measures.

### Statistical analysis

Data obtained will be analysed according to the intention-to-treat principle, such that each participant’s data will contribute to the findings for the group they were allocated to, irrespective of any subsequent diagnostic information or the treatment actually received. Reporting of the trial methodology and results will be according to the Consolidated Standards of Reporting Trials guidelines.^16^ The analysis will be prespecified in detail in a statistical analysis plan, which will be made publicly available prior to the trial team having access to the data. The findings for the primary outcome measure will be presented as an absolute difference in incidence between conservative management and control groups, with the limit of the one-sided 97.5% CI, compared with the non-inferiority bound of an absolute difference of a 7.5% higher incidence of the primary outcome in the conservative management group. The primary analysis will be based on the observed data, but the potential impact of any missing primary outcome measures on the trial conclusions will be investigated in sensitivity analyses. If non-inferiority is demonstrated, evidence from the risk difference, two-sided 95% CI and p value will be presented to allow inference about the superiority of conservative management compared with usual care.

### Health economic analysis

A cost-utility analysis with a maximal time horizon of 6 months (corresponding to the period of maximal follow-up for patient-reported pain, dyspnoea and mortality) will be undertaken, since this is the time period that clinicians and patient advisors advise us is long enough to capture all relevant effects. However, it is possible that we will see convergence of costs and outcomes within 30 days (which corresponds to the primary outcome), and, to explore this, we will report cost-effectiveness at both 30 days and 6 months.

The QALY will be derived by applying the cross-walk algorithm to the EQ-5D-5L^13^ and combining information on survival.

Resource use data are being collected on all NHS and personal social services care resources for trial participants up to 6 months. A patient-reported resource use questionnaire (note that the patient questionnaire will incorporate modular resource use measure,^17^ with the addition of some trial-specific questions) at 3 and 6 months will provide additional data on primary and community care resource after discharge.

Using medical notes from patients coded for chest drain insertion at one site, a set of assumptions has been established detailing staff, equipment, analgesia and imaging use relating to chest drain insertion and other high-cost pleural interventions in different settings, and these will be reviewed by clinicians at participating organisations for accuracy. The aim of this is to enhance our understanding of the trauma pathway and to inform and validate our costing approach.

### Qualitative analysis

The qualitative research aims to provide a comprehensive and in-depth understanding of the acceptability of initial conservative management versus chest drain to patients and clinicians by conducting interviews using topic guides; these guides are shown in online supplemental file 1. Patients and consultees will be approached at least 8 weeks after randomisation. Interviews will explore patient and consultee views and experiences of conservative management or chest drain insertion in the short to medium term, including impact on their daily life, positive and negative aspects of the treatment, pain, post-procedure recovery, subsequent treatments and return to activities. To enhance the understanding of clinician acceptability of initial conservative treatment and its implementation in wider practice, we will also interview clinicians involved in the trial patient pathway. Interviews will explore views of initial conservative management/chest drain, potential hidden benefits of initial conservative management, barriers to and facilitators for introducing initial conservative management into practice and what influences decision-making concerning traumatic pneumothorax management.

10.1136/bmjopen-2024-087464.supp1 Supplementary data

Maximum variation/purposive sampling will be used when possible, with the aim of achieving diversity in terms of participant characteristics. Anonymised transcripts will be analysed using reflexive thematic analysis.^18^ Transcripts will be coded for key categories and concepts, using deductive coding (based on the research aims and Theoretical Framework of Acceptability)^19^ and inductive coding (developing new codes based on issues emerging from the data) with the aid of NVivo software. Findings will be considered in relation to quantitative results and provide enhanced understanding of chest injury management in the emergency context.

### Safety

Participant safety will be monitored by the Trial Management Group (TMG), sponsor and oversight committees (Trial Steering Committee and Data Monitoring Committee). The protocol contains a list of events that can be expected in this patient population. If an expected serious adverse event (SAE) is prolonged or more serious than expected, this will be reported as an unexpected SAE.

All SAEs, expected non-serious adverse events (AEs) and non-serious AEs caused by pleural interventions (which occur up to 30 days post-randomisation for the latter) will be recorded in CRFs and monitored. SAEs that are both related to the trial (ie, resulted from conservative management or administration of a research procedure) and unexpected (ie, not listed in the protocol as expected) are suspected unexpected serious adverse reactions and will be subject to reporting to the sponsor and Research Ethics Committee (REC). We do not expect any AEs or SAEs related to conservative management (above those expected of the control arm, that is, standard care).

### Patient and public involvement

A PPI group made up of PPI co-applicants/members, and supplemented through networking and outreach work, meet as needed throughout the duration of the trial to ensure an iterative and responsive PPI strategy. Our PPI group have fed into all aspects of trial design, provided feedback on trial documents and have been involved in maximising retention of participants. A group of knife crime and violence reduction professionals from a boxing group in Bristol, Empire Fighting Chance, meet separately to address this important element of the trial.

### Trial management and oversight

The chief investigators take overall responsibility for managing the trial. Bristol Trials Centre, a UK Clinical Research Collaboration-registered trials unit, is responsible for the preparation of trial documents, site initiation visits and training, day-to-day running of the trial and monitoring of centres. The TMG oversees the trial and meets bimonthly to review progress. The Trial Steering Committee meets biannually to review conduct and progress and the Data Monitoring Committee at least annually to review data completion and safety. The trial sponsor is North Bristol NHS Trust, which oversees the trial and has ultimate responsibility for any decision about its continuation.

### Changes to trial protocol

Since the first trial protocol was approved by the REC (V.2.0, dated 11 May 2022), there have been four amendments to the protocol (current version is version 6.0, dated 23 February 2024). The first amendment (protocol version 3.0, 7 July 2022) clarified the eligibility criteria where bilateral pneumothoraces are present. The second amendment (protocol version 4.0, 15 December 2022) amended the key inclusion criterion from ‘in whom the treating clinician(s) are uncertain if a chest drain is required’ to ‘in whom the treating clinician(s) believes either a chest drain or conservative management is a suitable initial treatment option’, based on feedback from participating organisations. The third amendment (protocol version 5.0, 8 June 2023) allowed the recruitment of patients at NHS organisations in Scotland, via informed consent only, due to differences in legalities for patients judged not to have capacity in Scotland. The fourth amendment (protocol version 6.0, 23 February 2024) included the addition of a process whereby the central trial team contact the participant and offer to contact their GP to express any concerns, if a certain score is reached on the Impact of Events Scale questionnaire^14^.

## Discussion

This trial investigating initial conservative management of traumatic pneumothoraces is a pragmatic multicentre RCT aiming to establish whether this approach is non-inferior to chest drain insertion in terms of clinical and cost-effectiveness. Should an initial conservative approach prove non-inferior to invasive management, this is likely to lead to widespread changes in practice and reduce avoidable harm from chest drain insertion.

We recognise that this trial is both methodologically complex and will be a challenge to deliver in an emergency setting. The following aspects have been considered in order to ensure the trial can be successfully delivered and answer the aims and objectives.

### Clinical equipoise

Equipoise is the key to our third inclusion criterion which relates to whether the treating clinician(s) would feel comfortable treating a patient’s traumatic pneumothorax with a chest drain or conservative management. The subjective nature of this inclusion criterion has been our most significant challenge to date. During the initial stages of recruitment, this inclusion criterion read ‘in whom the treating clinician(s) are uncertain if a chest drain is required’. During the early stages of recruitment, both the trial team and the qualitative research team received feedback from site teams that clinicians may have been perceiving this as questioning their confidence in their clinical decision-making, rather than the intended ‘research uncertainty’, and that eligible patients may be being excluded due to this. An amendment was implemented to change this key inclusion criterion to ‘in whom the treating clinician(s) believes either a chest drain or conservative management is a suitable initial treatment option’. The trial team have emphasised when training site teams that a patient should be considered if the treating clinician acknowledges that the patient could be treated differently if seen by a colleague, or if they presented at a different NHS hospital. During training, case studies (anonymised radiology images) are shared with sites to illustrate the variation in the sizes of traumatic pneumothoraces within the trial, including mention of factors which affected decision-making (eg, presence of surgical emphysema, ventilation status, body mass index).

In addition, variation in embedded practice within the specialty groups involved in decision-making has been noticeable throughout the duration of the trial. Generally speaking, emergency clinicians seem more comfortable with treating small to moderate traumatic pneumothoraces conservatively, whereas there has been some reluctance from the surgical community, with a preference for chest drain insertion often observed. This may be due to concerns about the potential increased need for chest drain insertion on hospital wards and the resource available to do this. Site teams have been reassured that the number of patients recruited at each site will be relatively small, that only half of the patients will be allocated to the conservative management arm and, in addition, the need for subsequent intervention is likely to be low at ~1 in 10, based on previous observational data.^5^

### Recruitment of multiply injured patients

We anticipated that 40% of participants recruited would be intubated and ventilated, based on TARN data. However, in June 2023, analysis showed that only 8% of patients recruited were intubated and ventilated at the time of randomisation. This may have been due to a preference for invasive management in positively pressure-ventilated patients, despite prior evidence demonstrating that ventilation does not predict failure of conservative management.^5 10^ The TMG were concerned that this may affect the generalisability of the trial’s results. The trial team have been engaging the critical care community via infographics and webinars and have since seen an increase in the proportion of intubated and ventilated patients included to 15%.

### Trial information provision

Due to the heterogeneous target population, we have trial information material available in a variety of formats to facilitate maximal participation in eligible patients. We created a patient information video (https://comited.blogs.bristol.ac.uk/information-for-patients/) which was aimed towards younger people. Patients are able to provide consent to participate after watching the video, with a detailed patient information sheet also provided for further reading. Individuals from the charity Empire Fighting Chance made a valuable contribution towards creation of the patient information video, providing feedback and suggestions of ways to ensure the video was relevant to the target group. The video has received positive feedback from participating site research teams and has enabled at least one patient who was unable to read to participate in the trial.

There are two pathways via which to enrol a patient into the trial: obtaining informed consent from those with capacity or recruiting those lacking capacity (temporarily or permanently) under the emergency waiver of consent.^20^ In some cases, patients may initially seem alert (eg, be standing, walking or talking), and this may be mistaken for capacity to make an informed decision about participation in research, especially in those who are under the influence of alcohol or drugs (recreational or medication) or in extreme pain. We have found that, in some situations, such patients do not recall the ‘informed consent’ discussion when spoken to at a later date so we encourage site teams to keep this in mind and to enrol patients under the waiver of consent if they feel this is appropriate.

The CoMiTED research team, alongside collaborators, created a short video for those who were temporarily lacking capacity at the time they were admitted to the ED and enrolled under the waiver of consent (https://comited.blogs.bristol.ac.uk/for-patients-who-have-recovered-capacity/). The video explains that they were too unwell to be asked about taking part at the time a decision about their treatment needed to be made; therefore, doctors decided that it was safe for the patient to take part. The video explains that, now that the patient is well enough, they are being approached with details of the trial and being asked if they wish to provide consent for continued participation. The video is not specific to the CoMiTED trial and is available for use in all emergency care trials using the waiver of consent.

## Conclusion

The CoMiTED trial is a multicentre pragmatic RCT which has been designed to generate new evidence around the management of patients with traumatic pneumothoraces and has the potential to lead to significant changes in clinical practice.

### Trial status

**Figure 2:**
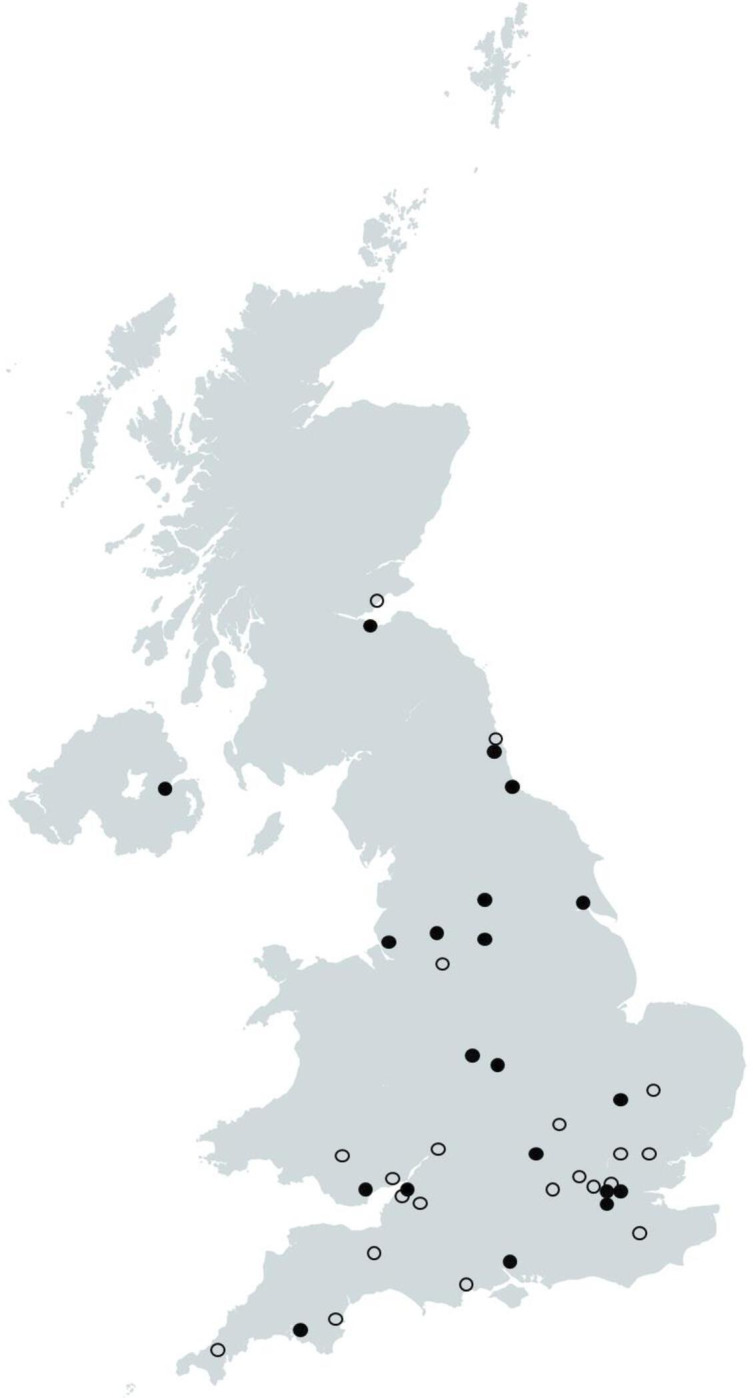
Distribution of participating organisations in the UK. Closed circles indicate major trauma centres and open circles indicate trauma units.

Recruitment to the trial began in August 2022, with an internal pilot to test feasibility. The trial is currently recruiting at 41 UK organisations (20 major trauma centres and 21 trauma units, distribution is shown in figure 2); and, as of 10 April 2024, 235 participants have been recruited across 35 sites.

### Ethics and dissemination

This trial was given a favourable opinion by the Wales REC 4 (reference: 22/WA/0118) and received approval from the Health Research Authority.

Trial participants are kept informed of trial progress via newsletters. A trial-specific X (previously Twitter) account, @CoMiTEDTrial, is used to promote the trial, provide updates and will disseminate findings. We aim to publish our primary, peer-reviewed manuscript in a high-impact medical journal and present our findings at multiple conferences. We will communicate our findings to the British Thoracic Society, National Institute for Health and Care Excellence and NHS England to incorporate the work into relevant national guidelines. The dataset will be published in the publicly available University of Bristol Research Data repository (https://www.bristol.ac.uk/staff/researchers/data/accessing-research-data/).

## Supplementary Material

Reviewer comments

Author's manuscript
