# Supplementary material for: Conservative management versus invasive management of significant traumatic pneumothoraces in the emergency department (the CoMiTED trial): a study protocol for a randomised non-inferiority trial
Source: BMJ Open. 2024 Jun 17;14(6):e087464. doi: 10.1136/bmjopen-2024-087464 (PMC11191772; doi:10.1136/bmjopen-2024-087464)
Supplement: Supplementary data [file bmjopen-2024-087464supp001.pdf]

CoMiTED

CoMiTED topic guides

Trial participant/consultee interviews

Background information on participant e.g., age, typical day, [consultees: – relationship to participant/carer responsibilities]

- **Experience leading to participation**
  - Can you tell me about how you/your relative (as appropriate) came to be admitted to the hospital? What happened? What were the injuries? If no memory of admittance – explore from when they can remember.
  - What happened to you/them after you/they were admitted to the hospital?

- **Trial views**
- Can you tell me about when you first heard about the CoMiTED study? Where, when, who?
  - **How was the study explained to you?**
  - Did you see a video about the study? What are your thoughts about the video? Good/bad/helpful?
- What did you think about the study? good idea/bad/any concerns?
- What is your understanding of the CoMiTED study? What are the researchers trying to do and why?
- Did the study make sense to you? probe why, why not – anything worried about?
- Could you tell me your thoughts on being involved in the study? Anything worried about? Anything they like about it?
  - How did feel about randomisation
  - If personal consultee, explore their understanding of their role and what is involved
  - Prompt paperwork/questionnaires
- Why did they decide to take part in the study/become a personal consultee?
- Thoughts on the consent process? Explore different routes
  - if were enrolled via deferred consent, explore their thoughts on this – understanding of/thoughts about?
- Why did you decide to take part (continue to take part if deferred consent)?
- Are you glad you took part in the study? Why, why not? Would you take part in the study again? Would you recommend family/friends to take part in the study? Explore.
- Questionnaires – any feedback, got on ok with them?
- Challenges/what could have improved your experience of taking part?
- Is there anything else you would have liked more information on?
- Are you glad you took part in the study? Why, why not? Would you do it again? Would you recommend family/friends to take part in the study? Explore. Explore whether and why views may have changed over time.

- **Treatment/symptom experiences**
- Explore treatments – chest drain/not when admitted – how long had drain for?
- What happened in the early days following the procedure/treatment, pain management, outcomes?
- Explore what they understand about the treatments (chest drain or not) they received and why they had it at the time of injury. Positive and negative aspects of the intervention, experience of

Blythe NM, *et al. BMJ Open* 2024; 14:e087464. doi: 10.1136/bmjopen-2024-087464

pain and its management, information provided, support available. Any subsequent medical visits/treatments? Experience of discharge.

- What was your experience of recovery following the procedure/treatment (since joining CoMiTED)?
- prompts: what happened after left hospital, earlier months – pain management, outcomes. Satisfaction with discharge? Were they told what to expect? Was this sufficient and met expectations?
- Describe any changes you have experienced in your symptoms up to now?
- prompt – duration, experiences of pain, breathlessness, functioning – changes in symptom experience, new onset symptoms?
- Have your symptoms changed much over the last few months (i.e. 3 vs 6 months)
- What symptoms are you experiencing now?
  - prompts – onset, duration, experiences of pain, breathlessness, functioning
- How do the symptoms bother you?
  - prompts – explore why bother them, what aspects of treatments more/less bothersome,
- How are symptoms affecting your daily life?
  - What impact has injury & treatment had on life
    - prompt – quality of life, personal costs e.g., sleep, social/other activities, emotional wellbeing, work, impact on family, relationships
    - Effect on self-perception, perception of others (e.g. scarring)
  - How have you been managing symptoms?
  - How long do they think the symptoms will last?
- What is their experiences of the treatments?
- Positive and negative aspects of the intervention, experience of pain and its management, information provided, support available. Any subsequent medical visits/treatments?

- **Final thoughts**
  - Thank you so much for your discussion. Do you have any final points that you would like to discuss or that you feel you didn't have the opportunity to talk about?

Health professional interviews

Background information e.g., HCP role, involvement in trial, years' experience,

Topics to be covered

i. Experiences of their participation in the trial

- How heard about the trial/why became involved in the trial
- Do you think there is a need for the CoMiTED study or not? Why?
- Which activities/processes been involved in – describe your experience of these

ii. Views, acceptability and decision-making for initial conservative management

- What is your normal decision-making process around using a chest drain/conservative management for traumatic pneumothoraces?
  - Prompts: What triggers a decision to treat, what informs your decision regarding treatment decisions – chest drain/initial conservative management, what are your typical treatment choices for people admitted to ED for traumatic pneumothoraces?

CoMiTED

- What are your thoughts/perspectives surrounding chest drain?
    - Prompts: perceptions regarding outcomes, appropriateness for which patients, procedure/technical considerations, when would you use it and why? Perceptions regarding symptom resolution, recovery from procedure, patient experiences, impact on quality of life.
- What are your thoughts/perspectives surrounding initial conservative management?
    - Prompts: perceptions regarding outcomes, appropriateness for which patients, procedure/technical considerations, when would you use it and why? Perceptions regarding symptom resolution, recovery from procedure, patient experiences, impact on quality of life.
- Have your perceptions changed surrounding the treatments being evaluated in CoMiTED throughout the study?
    - Prompts: patients may receive different treatments than usually advocated by clinician, have they been surprised by the outcomes, are outcomes as expected, how has the study altered their decision-making considerations, any different thoughts regarding the procedures compared to the start of the study?
- If the trial was to show a benefit to patients between the two different treatment approaches, how do you think this might alter your practice?
    - Prompts: what might you consider differently when deciding between chest drain and initial conservative management, would the findings persuade you to consider different options you may not have had before, what do you think the benefits of using initial conservative management within wider practice may be? Do you think this will be implementable, what might be the difficulties with implementing the trial findings in practice?

iii. Back to the trial

- How would you say CoMiTED has been going at your site? What have been the obstacles to getting going with CoMiTED?
- What has gone well? Any advice for other sites?
- How are decisions about whether the patient is eligible and should be entered into the trial made at your site? Who is involved? Have there been any issues? Overcome?
- How do you think having a multidisciplinary team approach to trauma care affects recruitment to the study? Difficulties? How these can be overcome?
- What are your thoughts on the eligibility criteria? Any issues?
- What might, in your opinion, improve running the trial at your site/other sites? (if not raised at this site, talk about how lack of equipoise has been an issue – any issue here_ How do they think this may be engaged with and tackled in CoMiTED?
- What are your thoughts on randomising patients within CoMiTED?
    - Appropriateness? Concerns? Patients responses?
- Are there instances when you didn't follow the randomisation allocation? Why? Explore reasons
- Views and experiences of the informed consent processes/different pathways including the videos
- Views and experiences of data collection (questionnaires/CRFs)
- Is there anything that we could do to improve about the study?

iv. Concluding interview

- Anything else to add, anything missed, important to capture?
- Thank them. Reassure of confidentiality
