# Supplementary material for: Conservative management versus invasive management of significant traumatic pneumothoraces in the emergency department (the CoMiTED trial): a study protocol for a randomised non-inferiority trial
Source: BMJ Open. 2024 Jun 17;14(6):e087464. doi: 10.1136/bmjopen-2024-087464 (PMC11191772; doi:10.1136/bmjopen-2024-087464)
Supplement: Author's manuscript [file bmjopen-2024-087464.draft_revisions.pdf]

BMJ Open

BMJ Open is committed to open peer review. As part of this commitment we make the peer review history of every article we publish publicly available.

When an article is published we post the peer reviewers' comments and the authors' responses online. We also post the versions of the paper that were used during peer review. These are the versions that the peer review comments apply to.

The versions of the paper that follow are the versions that were submitted during the peer review process. They are not the versions of record or the final published versions. They should not be cited or distributed as the published version of this manuscript.

BMJ Open is an open access journal and the full, final, typeset and author-corrected version of record of the manuscript is available on our site with no access controls, subscription charges or pay-per-view fees (http://bmjopen.bmj.com).

If you have any questions on BMJ Open's open peer review process please email info.bmjopen@bmj.com

BMJ Open

Conservative management versus invasive management of significant traumatic pneumothoraces in the Emergency Department (The CoMiTED Trial): a study protocol for a randomised non-inferiority trial

Journal:	*BMJ Open*
Manuscript ID	bmjopen-2024-087464
Article Type:	Protocol
Date Submitted by the Author:	10-Apr-2024
Complete List of Authors:	Blythe, Nicola; University of Bristol, Bristol Trials Centre<br>Coates, Katherine; North Bristol NHS Trust<br>Benger, Jonathan; University of the West of England<br>Annaw, Ammar; University of Bristol, Bristol Trials Centre<br>Banks, Jonathan; University of Bristol, Bristol Trials Centre<br>Clement, Clare; University of the West of England<br>Clout, Madeleine; University of Bristol, Bristol Trials Centre<br>Edwards, Antoinette; The University of Manchester<br>Gaunt, Daisy; University of Bristol, Bristol Trials Centre<br>Kandiyali, Rebecca; University of Warwick<br>Lane, Athene; University of Bristol, Bristol Trials Centre<br>Lecky, Fiona; The University of Sheffield; Salford Royal NHS Trust, Emergency Department<br>Maskell, Nick; University of Bristol, Academic Respiratory Unit, School of Clinical Sciences; North Bristol NHS Trust, Respiratory Research Unit<br>Metcalfe, Chris; University of Bristol, Bristol Trials Centre<br>Platt, Marie; University of Bristol, Bristol Trials Centre<br>Rees, Sophie; University of Bristol, Bristol Trials Centre<br>Taylor, Jodi; University of Bristol, Bristol Trials Centre<br>Thompson, Julian; North Bristol NHS Trust<br>Walker, Steven; University of Bristol Academic Respiratory Unit<br>West, Douglas; University Hospitals Bristol and Weston NHS Foundation Trust<br>Carlton, Edward; University of Bristol; North Bristol NHS Trust, Emergency Department
Keywords:	ACCIDENT & EMERGENCY MEDICINE, Randomized Controlled Trial, TRAUMA MANAGEMENT

SCHOLARONE™
Manuscripts

Conservative management versus invasive management of significant traumatic pneumothoraces in the Emergency Department (The CoMiTED Trial): a study protocol for a randomised non-inferiority trial

Nicola M Blythe, Katherine Coates, Jonathan Benger, Ammar Annaw, Jonathan Banks, Clare Clement, Madeleine Clout, Antoinette Edwards, Daisy Gaunt, Rebecca Kandiyali, J Athene Lane, Fiona Lecky, Nick Maskell, Chris Metcalfe, Marie Platt, Sophie Rees, Jodi Taylor, Julian Thompson, Steven Walker, Douglas West and Edward Carlton.

Word count: 4122 (excluding title page, abstract, figures and tables, acknowledgements, contributions and references)

Number of tables: 2

Number of figures: 2 (submitted separately)

Corresponding author: Professor Edward Carlton, Emergency Department, Southmead Hospital, North Bristol NHS Trust, Southmead Road, Bristol, BS10 5NB. Email: ed.carlton@bristol.ac.uk.

Key words: accident and emergency medicine, trauma management, randomised controlled trial, traumatic pneumothorax, conservative management

For peer review only

ABSTRACT

Introduction

Traumatic pneumothoraces are present in 1 in 5 victims of severe trauma. Current guidelines advise chest drain insertion for most traumatic pneumothoraces, although very small pneumothoraces can be managed with observation at the treating clinician's discretion. There remains a large proportion of patients in whom there is clinical uncertainty as to whether an immediate chest drain is required, with no robust evidence to inform practice. Chest drains carry a high risk of complications such as bleeding and infection. The default to invasive treatment may be causing potentially avoidable pain, distress and complications. We are evaluating the clinical and cost-effectiveness of an initial conservative approach to the management of patients with traumatic pneumothoraces.

Methods and analysis

The CoMiTED trial is a multicentre, pragmatic parallel group, individually randomised controlled non-inferiority trial to establish whether initial conservative management of significant traumatic pneumothoraces is non-inferior to invasive management in terms of subsequent emergency pleural interventions, complications, pain, breathlessness, and quality of life. We aim to recruit 750 patients from at least 40 UK NHS hospitals. Patients allocated to the control (invasive management) group will have a chest drain inserted in the emergency department. For those in the intervention (initial conservative management) group, the treating clinician will be advised to manage the participant without chest drain insertion and undertake observation. The primary outcome is a binary measure of the need for one or more subsequent emergency pleural interventions within 30 days of randomisation. Secondary outcomes include complications, cost-effectiveness, patient-reported quality of life and patient and clinician views of the two treatment options; participants are followed up for 6 months.

Ethics and dissemination

This trial received approval from Wales Research Ethics Committee 4 (reference: 22/WA/0118) and the Health Research Authority. Results will be submitted for publication in a peer-reviewed journal.

Trial registration number ISRCTN35574247.

Strengths and Limitations of this study

- This is a pragmatic trial; once the initial decision has been made and patients have been allocated a treatment arm, all subsequent care and interventions are at the discretion of treating clinical teams.
- Patients can be recruited from the whole of the trauma spectrum, from those with isolated chest injuries to victims of polytrauma. This will include patients with varying injury mechanisms (e.g. penetrating and blunt trauma), patients receiving positive pressure ventilation and older patients living with frailty, to ensure results can be generalisable across the diverse trauma population.
- The trial involves economic evaluation and has an integrated qualitative study, in order to determine the clinical and cost effectiveness of initial conservative management versus invasive management of traumatic pneumothoraces and to assess the acceptability of initial conservative management to patients and clinicians, respectively.
- Blinding to treatment allocation is not possible for clinicians or participants; only clinicians adjudicating primary outcome and researchers evaluating outcomes for the analyses will be blinded to treatment group.

INTRODUCTION

Injury is a leading cause of death among adults aged <45 years [1]. Traumatic pneumothoraces are present in 1 in 5 victims of severe trauma [2, 3]. We estimate from prior observational and survey work [4, 5] that around half of patients admitted to hospital with traumatic pneumothoraces will be treated with the insertion of a chest drain. Current guidelines advise chest drain insertion for most traumatic pneumothoraces, although very small pneumothoraces can be managed with observation at the treating clinician's discretion [6, 7]. For some patients with very large pneumothoraces, chest drain placement can reduce the risk of cardiorespiratory compromise [8]. However, there remains a large proportion of patients in whom there is clinical uncertainty as to whether an immediate chest drain is required [4]. Chest drains carry a high-risk of complications, such as bleeding and infection, in 15-30% of patients [9]. There is a lack of robust evidence to inform practice, and the default to invasive treatment may cause potentially avoidable patient harm.

In an analysis of >600 patients with traumatic pneumothoraces from 2012 to 2016, obtained from Trauma Audit & Research Network (TARN) data, 90% of patients treated without a chest drain did not require subsequent intervention [5], suggesting a potential role for conservative management. However, in this analysis, 50% of patients were initially treated with a chest drain and there was considerable clinical variation in those selected for this invasive procedure. In a 2020 international survey of 222 emergency physicians [4], utilising clinical vignettes of larger traumatic pneumothoraces, over 60% of clinicians would elect to insert a chest drain in the Emergency Department (ED), even without clinical compromise. Therefore, based on the observational studies and lack of robust data, we designed a randomised controlled trial (RCT) to assess the clinical and cost-effectiveness of an initial conservative approach to the management of patients with traumatic pneumothoraces. If we demonstrate that this approach achieves similar clinical outcomes, it will reduce the use of a painful, invasive and potentially harmful management strategy.

For peer review only - http://bmjopen.bmj.com/site/about/guidelines.xhtml

Prior to the start of the trial, we searched Medline for systematic reviews, and Medline, Embase, Cochrane Central, ClincalTrials.gov and the World Health Organisation (WHO) trials registry for trials published. One systematic review from 2010 evaluated three small (total n=101) RCTs examining the safety of conservative management in small traumatic pneumothoraces [8]. This review suggested that conservative management may be at least as safe and effective as chest drain insertion. A further multicentre RCT of small pneumothoraces in severely injured patients in Canada concluded in 2021 [10]. These patients (142 in total) were all receiving positive pressure ventilation and current guidelines suggest chest drain insertion in all patients undergoing ventilation [2, 4]. The results showed no difference in mortality or intensive care unit (ICU) or hospital length of stay between patients who were conservatively managed and those who had chest drains inserted. The authors concluded that small traumatic pneumothoraces may be safely observed in patients undergoing ventilation and that the complications of chest drains remain unacceptably high. By including only patients undergoing ventilation (which is around 30% of the traumatic pneumothorax population in the UK [5]), the Canadian study did not fully address conservative management in the broader trauma population, as we are in this trial.

Aims and objectives

The Conservative Management in Traumatic Pneumothoraces in the Emergency Department (CoMiTED) trial will test whether initial conservative management of significant traumatic pneumothoraces is non-inferior to invasive management in terms of subsequent emergency pleural interventions, complications, pain, breathlessness, and quality of life.

Specific objectives are:

a) To establish if initial conservative management is non-inferior to invasive management regarding subsequent emergency pleural intervention over 30 days (or until death if sooner).

b) To determine whether conservative management improves health-related quality of life and other patient reported outcomes.

c) To determine the clinical and cost effectiveness of initial conservative management versus invasive management of traumatic pneumothoraces by measuring resource use, mortality and costs over the six months following injury.

d) To assess acceptability of initial conservative management to patients and clinicians.

METHODS AND ANALYSIS

Trial design

The CoMiTED trial is a pragmatic multicentre, parallel group, individually randomised controlled non-inferiority trial with an economic evaluation and integrated qualitative study.

Setting

The trial will recruit patients from approximately 40 NHS Major Trauma Centres and Trauma Units across the UK.

For peer review only - http://bmjopen.bmj.com/site/about/guidelines.xhtml

Trial population

Inclusion and exclusion are detailed in Table 1.

Inclusion Criteria	Exclusion Criteria
Presenting with traumatic pneumothorax/pneumothoraces	Treating clinician(s) believes injuries are incompatible with life
(Believed to be) 16 years and over	Patient in respiratory arrest
Treating clinician(s) believes either a chest drain or conservative management is a suitable initial treatment option	Haemothorax (associated with pneumothorax) requiring a chest drain in the opinion of the treating clinician(s)*
	Clinical or imaging evidence of tension pneumothorax in either lung at the point of randomisation
	Prisoner

Table 1. Table illustrating inclusion and exclusion criteria. Special Circumstances: In patients presenting with bilateral chest injury, if one lung of the patient qualifies, the patient can be enrolled, providing no exclusion criteria are met. Treatment of the eligible side follows the randomisation assignment, with the other side treated according to usual practice. If both sides qualify, both sides receive treatment according to the randomisation assignment. Patients who have received pre-hospital thoracostomies may still be enrolled, provided they fulfil the eligibility criteria. Where a participant who has received a prehospital thoracostomy is randomised to conservative management, local practice should be followed.

**Patients with an associated haemothorax are excluded due to this being a predictor of failure of conservative management [5].*

Primary outcome

The primary outcome is a binary measure of the need for one or more subsequent emergency pleural interventions in the eligible lung(s), from the point of randomisation up to 30 days. This excludes chest drain insertion in the ED for those allocated to the chest drain (control) group.

Reasons for subsequent emergency chest drain insertion may include (but are not limited to): clinically significant symptoms persisting despite adequate analgesia; chest pain or breathlessness preventing activity; a patient is unwilling to continue with conservative treatment; the patient's condition becomes physiologically unstable presumed secondary to pneumothorax; repeat chest radiograph shows an enlarging pneumothorax with physiological instability. Reasons for subsequent emergency pleural intervention are determined by local practice and recorded but are not controlled.

Whether a subsequent pleural intervention is deemed to be an emergency is adjudicated by a panel made up of independent expert clinicians from relevant specialties. The clinicians are blinded to allocation and presented with clinical and imaging vignettes of what happened to each participant and subsequently asked to determine whether, in their opinion, any subsequent pleural intervention that occurred within 30 days of randomisation was required due to an emergency. Consensus agreement is obtained by two members of the panel.

For peer review only - http://bmjopen.bmj.com/site/about/guidelines.xhtml

For peer review only

Secondary outcomes

The secondary outcomes will capture any differences between the allocated groups in terms of reduced pain, complications and improved health-related quality of life in the short to medium term, as well as inform a formal cost-effectiveness analysis.

Secondary outcomes are as follows: (i) All pleural interventions (including chest drain insertion in the ED) up to 30 days, (ii) All complications of pleural intervention up to 30 days, (iii) Total days of pleural drainage up to 30 days, (iv) Patient-reported pain [11], function and breathlessness [12] at baseline, 30 days, 3 and 6 months, (v) Quality of life [13, 14] at baseline, 30 days, 3 and 6 months, (vi) Total length of stay (hospital, critical care (including HDU) admission and readmission) up to 30 days, (vii) Adjudicated mortality at 30 days (pneumothorax or chest injury related), (viii) All-cause mortality at 6 months, (iv) Cost per quality-adjusted life year (QALY) at 6 months, (x) Patient views and experiences of conservative management/chest drain and (xi) Clinician views of conservative management/chest drain. For this trial, baseline patient reported outcome measures (PROMs) can be collected from as soon as feasible following randomisation and after treatment delivery (where appropriate) up to 7 days post-randomisation.

Sample size

We aim to recruit 750 participants and conduct approximately 25 patient interviews for the integrated qualitative study over 37 months (August 2022 – September 2025).

Observational data suggests that 10% of our trial population will require emergency pleural intervention following conservative management [5]. Our group recently identified a reintervention rate of 10% following initial chest drain insertion in a single UK site [5] and therefore anticipate the incidence of the primary outcome in the control group to be at least 10%.

Our patient and public involvement (PPI) contributors unanimously support the potential advantages of initial conservative management, such as avoiding an invasive procedure, improved mobilisation after injury, and reduced longer term pain. However, they also recognise the need to balance these benefits against the risk of avoidable harm. When asked, our PPI group felt that an increase of 5-10% in subsequent emergency pleural intervention would be acceptable compared to usual care, given the anticipated reduction in the overall number of chest drains in the intervention group. These views have been used to select a non-inferiority margin of 7.5%. We will conclude that the trial population can be safely managed conservatively if the incidence of subsequent emergency pleural intervention is no more than 7.5% higher in the intervention group than in the control group. If the incidence of the primary outcome is 10% in both groups, a sample size of 674 (337 in each group) will allow non-inferiority to be concluded with 90% power, when comparing a one-sided 97.5% confidence interval, for the absolute difference in primary outcome incidence, to a non-inferiority margin of 7.5%. Allowing 10% loss to follow-up increases the total sample size to 750.

Patient approach, recruitment and randomisation

Following eligibility assessment, eligible patients undergo a capacity assessment. If the patient has capacity, they are approached in the ED for their consent to take part. Where patients are judged to be unable to provide informed consent for themselves, then patients can be automatically enrolled under the waiver of consent (in countries where the waiver of consent is permitted for non-CTIMP trials). If patients regain capacity within 7 days post-randomisation, they are approached and asked whether they wish to provide consent to continue in the trial. If patients do not regain capacity

For peer review only - http://bmjopen.bmj.com/site/about/guidelines.xhtml

within 7 days of randomisation, a member of the research team seeks advice from a Personal Consultee or, if unavailable, a Nominated Consultee. The participant pathway is shown in Figure 1.

Patients are randomised in the ED immediately after traumatic pneumothorax has been diagnosed and consent provided / waiver of consent applied. Participants are allocated in a 1:1 ratio to either "chest drain insertion in the ED (control group)" or "initial conservative management (intervention group)" using a secure web-based randomisation system (Sealed Envelope, https://www.sealedenvelope.com/). Randomised allocation is minimised by three factors: 'trial site', 'currently ventilated' and 'penetrating injury'.

Trial intervention

In the intervention (initial conservative management) group, the treating clinician is advised to manage the participant without chest drain insertion and undertake observation and admission to a hospital ward or ICU. Given the pragmatic nature of this trial, all subsequent interventions and further imaging to evaluate pneumothorax resolution after the point of randomisation, is at the discretion of the treating clinical teams.

In the control group (chest drain insertion in the ED), the treating clinician is advised to insert a chest drain. Specific details of the procedure (including anaesthesia and insertion technique) is at the discretion of treating clinicians but will be recorded for trial purposes.

Data collection

Trial data is collected at baseline, 30 days, 3 months and 6 months and recorded by participating site team members onto case report forms (CRFs) and participant-completed questionnaires. Table 2 depicts the key assessments/outcome measures and participant-related procedures scheduled at various trial timepoints. These are entered into a REDCap database [15] for data cleaning and analysis. Access to the database is via a secure password-protected web interface.

**Data collection timepoint (→)**	**In the Emergency Department (ED)**		**Post-Randomisation Follow Ups**		
**Data capture / key trial procedure (↓)**	**Recruitment (Day 0)**	**Post-Recruitment (Baseline)**	**30 days**	**3 months**	**6 months**
Screening, Consent & Randomisation	X				
Case Report Form including safety reporting (CRF)	X	X	X	X	X
Sociodemographic Details		X			
Injury Details		X			
Injury Severity Score		X			
Comorbidities		X			

For peer review only - http://bmjopen.bmj.com/site/about/guidelines.xhtml

National Early Warning Score (routinely collected)		X			
PROMS; Pain (Brief Pain Inventory)		X	X	X	X
PROMS; Function (Brief Pain Inventory)		X	X	X	X
PROMS; Breathlessness (MRC dyspnoea scale)		X	X	X	X
PROMS; Quality of life (EQ-5D-5L)		X	X	X	X
PROMS; Impact Events Scale (IES-R)			X	X	X
Patient completed resource use questionnaire				X	X
Pleural interventions			X		
Complications			X		
Days of pleural drainage			X		
Length of stay (hospital and critical care (including HDU) admission, and readmission)			X		
Details of re-attendances to A&E or unplanned re-admissions within 30 days			X		
Mortality			X		X
Qualitative interviews			X		X

Table 2. Schedule of essential data capture and participant-related procedures.

For peer review only

Statistical analysis

Data obtained will be analysed according to the intention to treat principle, such that each participant's data will contribute to the findings for the group they were allocated to, irrespective of any subsequent diagnostic information or the treatment actually received. Reporting of the trial methodology and results will be according to the CONSORT guidelines [16]. The analysis will be pre-specified in detail in a statistical analysis plan, which will be made publicly available prior to the trial team having access to the data. The findings for the primary outcome measure will be presented as an absolute difference in incidence between conservative management and control groups, with the limit of the one-sided 97.5% confidence interval, compared to the non-inferiority bound of an absolute difference of a 7.5% higher incidence of the primary outcome in the conservative

For peer review only - http://bmjopen.bmj.com/site/about/guidelines.xhtml

management group. The primary analysis will be based on the observed data, but the potential impact of any missing primary outcome measures on the trial conclusions will be investigated in sensitivity analyses. If non-inferiority is demonstrated, evidence from the risk difference, two-sided 95% confidence interval, and p-value, will be presented to allow inference about the superiority of conservative management compared to usual care.

Health economic analysis

A cost utility analysis with a maximal time horizon of 6 months (corresponding to the period of maximal follow-up for patient-reported pain, dyspnoea and mortality) will be undertaken, since this is the time period that clinicians and patient advisors advise us is long enough to capture all relevant effects. However, it is possible that we will see convergence of costs and outcomes within 30 days (which corresponds to the primary outcome), and, to explore this, we will report cost-effectiveness at both 30 days and 6 months.

The QALY will be derived by applying the cross-walk algorithm to the EQ-5D-5L [13] and combining information on survival.

Resource use data is being collected on all NHS and personal social services care resources for trial participants up to 6 months. A patient-reported resource use questionnaire (note that the patient questionnaire will incorporate ModRUM [17], with the addition of some trial-specific questions) at 3 and 6 months will provide additional data on primary and community care resource after discharge.

Utilising medical notes from patients coded for chest drain insertion at one site, a set of assumptions have been established detailing staff, equipment, analgesia and imaging use relating to chest drain insertion and other high-cost pleural interventions in different settings and these will be reviewed by clinicians at participating organisations for accuracy. The aim of this is to enhance our understanding of the trauma pathway and to inform and validate our costing approach.

Qualitative analysis

The qualitative research aims to provide a comprehensive and in-depth understanding of the acceptability of initial conservative management versus chest drain to patients and clinicians by conducting interviews using topic guides. Patients and consultees will be approached at least eight weeks after randomisation. Interviews will explore patient and consultee views and experiences of conservative management or chest drain insertion in the short to medium term, including impact on their daily life, positive and negative aspects of the treatment, pain, post-procedure recovery, subsequent treatments and return to activities. To enhance the understanding of clinician acceptability of initial conservative treatment and its implementation in wider practice, we will also interview clinicians involved in the trial patient pathway. Interviews will explore views of initial conservative management/chest drain, potential hidden benefits of initial conservative management, barriers to and facilitators for introducing initial conservative management into practice and what influences decision-making concerning traumatic pneumothorax management.

Maximum variation/purposive sampling will be used when possible, with the aim of achieving diversity in terms of participant characteristics. Anonymised transcripts will be analysed using reflexive thematic analysis [18]. Transcripts will be coded for key categories and concepts, using deductive coding (based on the research aims and Theoretical Framework of Acceptability) [19] and inductive coding (developing new codes based on issues emerging from the data) with the aid of NVivo software. Findings will be considered in relation to quantitative results and provide enhanced understanding of chest injury management in the emergency context.

For peer review only - http://bmjopen.bmj.com/site/about/guidelines.xhtml

Safety

Participant safety will be monitored by the Trial Management Group (TMG), Sponsor and oversight committees (Trial Steering Committee and Data Monitoring Committee). The protocol contains a list of events that can be expected in this patient population. If an expected serious adverse event (SAE) is prolonged or more serious than expected, this will be reported as an unexpected SAE.

All SAEs, expected non-serious adverse events (AEs) and non-serious AEs caused by pleural interventions (which occur up to 30 days post-randomisation for the latter) will be recorded in CRFs and monitored. SAEs that are both related to the trial (i.e. resulted from conservative management or administration of a research procedure) and unexpected (i.e. not listed in the protocol as expected) are suspected unexpected serious adverse reactions (SUSARs) and will be subject to reporting to the Sponsor and Research Ethics Committee (REC). We do not expect any AEs or SAEs related to conservative management (above those expected of the control arm, i.e. standard care).

Patient and Public Involvement

A PPI group made up of PPI co-applicants/members, and supplemented through networking and outreach work, meet as needed throughout the duration of the trial to ensure an iterative and responsive PPI strategy. Our PPI group have fed into all aspects of trial design, provided feedback on trial documents and have been involved in maximising retention of participants. A group of knife crime and violence reduction professionals from a boxing group in Bristol, Empire Fighting Chance, meet separately to address this important element of the trial.

Trial management and oversight

The Chief Investigators take overall responsibility for managing the trial. Bristol Trials Centre, a UK Clinical Research Collaboration-registered trials unit, is responsible for the preparation of trial documents, site initiation visits and training, day-to-day running of the trial and monitoring of centres. The TMG oversees the trial and meets bimonthly to review progress. The trial steering committee meets biannually to review conduct and progress and the data monitoring committee at least annually to review data completion and safety. The trial Sponsor is North Bristol NHS Trust, who oversees the trial and has ultimate responsibility for any decision about its continuation.

Changes to trial protocol

Since the first trial protocol was approved by the Research and Ethics Committee (V2.0, dated 11 May 2022), there have been three amendments to the protocol (current version is version 5.0, dated 08 June 2023). The first amendment (protocol version 3.0, 07 July 2022) clarified the eligibility criteria where bilateral pneumothoraces are present. The second amendment (protocol version 4.0, 15 December 2022), amended the key inclusion criterion from '*in whom the treating clinician(s) are uncertain if a chest drain is required*' to '*in whom the treating clinician(s) believes either a chest drain or conservative management is a suitable initial treatment option*', based on feedback from participating organisations. The third amendment, (protocol version 5.0, 08 June 2023), allowed the recruitment of patients at NHS organisations in Scotland, via informed consent only, due to differences in legalities for patients judged not to have capacity in Scotland.

For peer review only - http://bmjopen.bmj.com/site/about/guidelines.xhtml

DISCUSSION

This trial investigating initial conservative management of traumatic pneumothoraces is a pragmatic multicentre RCT aiming to establish whether this approach is non-inferior to chest drain insertion in terms of clinical and cost-effectiveness. Should an initial conservative approach prove non-inferior to invasive management, this is likely to lead to widespread changes in practice and reduce avoidable harm from chest drain insertion.

We recognise that this trial is both methodologically complex and will be a challenge to deliver in an emergency setting. The following aspects have been considered in order to ensure the trial can be successfully delivered and answer the aims and objectives.

Clinical Equipoise

Equipoise is the key to our third inclusion criterion which relates to whether the treating clinician(s) would feel comfortable treating a patient's traumatic pneumothorax with a chest drain or conservative management. The subjective nature of this inclusion criterion has been our most significant challenge to date. During the initial stages of recruitment, this inclusion criterion read '*in whom the treating clinician(s) are uncertain if a chest drain is required*'. During the early stages of recruitment, both the trial team and the qualitative research team received feedback from site teams that clinicians may have been perceiving this as questioning their confidence in their clinical decision-making, rather than the intended 'research uncertainty', and that eligible patients may be being excluded due to this. An amendment was implemented to change this key inclusion criterion to '*in whom the treating clinician(s) believes either a chest drain or conservative management is a suitable initial treatment option*'. The trial team have emphasised when training site teams that a patient should be considered if the treating clinician acknowledges that the patient could be treated differently if seen by a colleague, or if they presented at a different NHS hospital. During training, case studies (anonymised radiology images) are shared with sites to illustrate the variation in the sizes of traumatic pneumothoraces within the trial, including mention of factors which affected decision-making (e.g. presence of surgical emphysema, ventilation status, body mass index).

In addition, variation in embedded practice within the specialty groups involved in decision-making has been noticeable throughout the duration of the trial. Generally speaking, emergency clinicians seem more comfortable with treating small to moderate traumatic pneumothoraces conservatively, whereas there has been some reluctance from the surgical community, with a preference for chest drain insertion often observed. This may be due to concerns about the potential increased need for chest drain insertion on hospital wards and the resource available to do this. Site teams have been reassured that the number of patients recruited at each site will be relatively small, that only half of the patients will be allocated to the conservative management arm and, in addition, the need for subsequent intervention is likely to be low at ~1 in 10, based on previous observational data [5].

Recruitment of multiply-injured patients

We anticipated that 40% of participants recruited would be intubated and ventilated, based on TARN data. However, in June 2023, analysis showed that only 8% of patients recruited were intubated and ventilated at the time of randomisation. This may have been due to a preference for invasive management in positively pressure ventilated patients, despite prior evidence demonstrating that ventilation does not predict failure of conservative management [5,10]. The TMG were concerned that this may affect the generalisability of the trial's results. The trial team

For peer review only - http://bmjopen.bmj.com/site/about/guidelines.xhtml

have been engaging the critical care community via infographics and webinars and have since seen an increase in the proportion of intubated and ventilated patients included to 15%.

Trial information provision

Due to the heterogenous target population, we have trial information material available in a variety of formats to facilitate maximal participation in eligible patients. We created a patient information video (https://comited.blogs.bristol.ac.uk/information-for-patients/) which was aimed towards younger people. Patients are able to provide consent to participate after watching the video, with a detailed patient information sheet also provided for further reading. Individuals from the charity Empire Fighting Chance made a valuable contribution towards creation of the patient information video, providing feedback and suggestions of ways to ensure the video was relevant to the target group. The video has received positive feedback from participating site research teams and has enabled at least one patient who was unable to read to participate in the trial.

There are two pathways via which to enrol a patient into the trial: obtaining informed consent from those with capacity or recruiting those lacking capacity (temporarily or permanently) under the emergency waiver of consent [20]. In some cases, patients may initially seem alert (e.g. be standing, walking or talking), and this may be mistaken for capacity to make an informed decision about participation in research, especially in those who are under the influence of alcohol or drugs (recreational or medication) or in extreme pain. We have found that, in some situations, such patients do not recall the 'informed consent' discussion when spoken to at a later date so we encourage site teams to keep this in mind and to enrol patients under the waiver of consent if they feel this is appropriate.

The CoMiTED research team, alongside collaborators, created a short video for those who were temporarily lacking capacity at the time they were admitted to the ED and enrolled under the waiver of consent (https://comited.blogs.bristol.ac.uk/for-patients-who-have-recovered-capacity/). The video explains that they were too unwell to be asked about taking part at the time a decision about their treatment needed to be made, therefore doctors decided that it was safe for the patient to take part. The video explains that, now that the patient is well enough, they are being approached with details of the trial and being asked if they wish to provide consent for continued participation. The video is not specific to the CoMiTED trial and is available for use in all emergency care trials utilising the waiver of consent.

For peer review only

Trial status

Recruitment to the trial began in August 2022, with an internal pilot to test feasibility. The trial is currently recruiting at 41 UK organisations (20 major trauma centres and 21 trauma units, distribution is shown in Figure 2); and, as of 10/04/2024, 235 participants have been recruited across 35 sites.

Conclusion

The CoMiTED trial is a multicentre pragmatic RCT which has been designed to generate new evidence around the management of patients with traumatic pneumothoraces and has the potential to lead to significant changes in clinical practice.

For peer review only - http://bmjopen.bmj.com/site/about/guidelines.xhtml

Ethics and dissemination

This trial was given a favourable opinion by Wales Research Ethics Committee 4 (reference: 22/WA/0118) and received approval from the Health Research Authority.

Trial participants are kept informed of trial progress via newsletters. A trial-specific X (previously Twitter) account, @CoMiTEDTrial, is used to promote the trial, provide updates and will disseminate findings. We aim to publish our primary, peer-reviewed manuscript in a high impact medical journal and present our findings at multiple conferences. We will communicate our findings to the British Thoracic Society, National Institute for Health and Care Excellence and NHS England to incorporate the work into relevant national guidelines. The dataset will be published in the publicly available University of Bristol Research Data repository https://www.bristol.ac.uk/staff/researchers/data/accessing-research-data/.

Acknowledgments

The authors thank all of the research and clinical team members at participating sites, members of the independent Trial Steering Committee, Data Monitoring Committee and adjudication panel, members of the patient and public involvement group and all of the participants who have taken part in this research so far. The authors would also like to thank Kate Beckett, Holly Mckeon and Amanda Lewis for their contributions.

Contributors

EC and JB are the Co-Chief Investigators, identified the funding opportunity and designed the trial, alongside co-applicants. NB set-up and manages the day-to-day running of the trial and assembled the manuscript from the trial protocol. KC and MP manage the day-to-day running of the trial. CM supervises the statistical analysis, and will write the statistical analysis plan. DG provides progress reports to the Trial Management Group and the independent oversight committees, and will complete the statistical analysis. SR leads the qualitative research, alongside JBa and, previously, CC. RK leads the health economics research, alongside AA. JT, AL & MC provide senior trial management oversight and advice. AE, FL, NM, SW, JTh and DW provide advice as part of the Trial Management Group. All authors were involved in preparation of the trial protocol and have read and approved the final manuscript.

Funding

The project is funded by the National Institute for Health and Care Research (NIHR) Health Technology Assessment Programme (HTA) (ref: NIHR132889). The views expressed are those of the authors and not necessarily those of the NIHR or the Department of Health and Social Care. This trial has been designed and delivered in collaboration with Bristol Trials Centre, a UK Clinical Research Collaboration-registered clinical trials unit, which is in receipt of NIHR clinical trials unit support funding.

Competing interests

The authors declare that they have no competing interests.

For peer review only

References

1. Moran C, Lecky F, Bouamra O, et al. Changing the System – Major Trauma Patients and Their Outcomes in the NHS (England) 2008–17. Lancet Eclinical Medicine. 2018;2:13-21.
2. Di Bartolomeo S, Sanson G, Nardi G, et al. A population-based study on pneumothorax in severely traumatized patients. J Trauma. 2001;51(4):677-82.
3. Kshettry VR, Bolman RM, 3rd. Chest trauma. Assessment, diagnosis, and management. Clin Chest Med. 1994;15(1):137-46.
4. Avery P, Watkins M, Benger J, et al. Current management of moderate to severe traumatic pneumothoraces: a survey of emergency clinicians. Emerg Med J. 2021.
5. Walker SP, Barratt SL, Thompson J, et al. Conservative Management in Traumatic Pneumothoraces: An Observational Study. Chest. 2018;153(4):946-53.
6. Surgeons Aco. Advanced Trauma Life Support® Student Course Manual. 10th Edition ed:Chicago: American College of Surgeons; 2018.
7. Thies KMG, P. European Trauma Course 4th edition. 2016.
8. Yadav K, Jalili M, Zehtabchi S. Management of traumatic occult pneumothorax. Resuscitation. 2010;81(9):1063-8.
9. Menger R, Telford G, Kim P, et al. Complications following thoracic trauma managed with tube thoracostomy. Injury. 2012;43(1):46-50.
10. Clements TW, Sirois M, Parry N, et al. OPTICC: A multicentre trial of Occult Pneumothoraces subjected to mechanical ventilation: The final report. American journal of surgery. 2021;221(6).
11. Tan G, Jensen MP, Thornby JI, at al. Validation of the Brief Pain Inventory for chronic nonmalignant pain. J Pain. 2004;5(2):133-7.
12. Stenton C. The MRC breathlessness scale. Occupational Medicine. 2008;58(3):226-7.
13. EuroQol G. EuroQol--a new facility for the measurement of health-related quality of life. Health Policy. 1990;16(3):199-208.
14. Weiss DS, Marmar C, Wilson JP, et al. Assessing psychological trauma and PTSD. 1997.
15. Harris PA, Taylor R, Thielke R, et al. Research electronic data capture (REDCap)–a metadata-driven methodology and workflow process for providing translational research informatics support. J Biomed Inform. 2009;42(2):377–81.
16. CONSORT Group. Consort transparent reporting of trials, 2010. Available: http://www. consort- statement.org/
17. Garfield K, Husbands S, Thorn JC, et al. Development of a brief, generic, modular resource-use measure (ModRUM): cognitive interviews with patients. BMC Health Services Research. 2021;21(1):1-12.
18. Braun V, Clarke V. Reflecting on reflexive thematic analysis. Qualitative Research in Sport, Exercise and Health. 2019;11(4):589-97.
19. Sekhon M, Cartwright M, Francis JJ. Acceptability of healthcare interventions: an overview of reviews and development of a theoretical framework. BMC Health Serv Res. 2017;17(1):88.
20. Health Research Authority (HRA). Principles of consent: Emergency Research (England and Wales) [19 November 2021]. Available from: http://www.hradecisiontools.org.uk/consent/principles-emergency-EnglandandWales.html.

For peer review only - http://bmjopen.bmj.com/site/about/guidelines.xhtml

Figure legends (figures submitted separately)

Figure 1. Trial schema illustrating the pathway for CoMiTED participants.

**It should be noted that patients without capacity are not being recruited in Scotland (Pathway B in England, Wales and Northern Ireland only).*

Figure 2. Distribution of participating organisations in the UK. Closed circles indicate major trauma centres and open circles indicate trauma units.

For peer review only

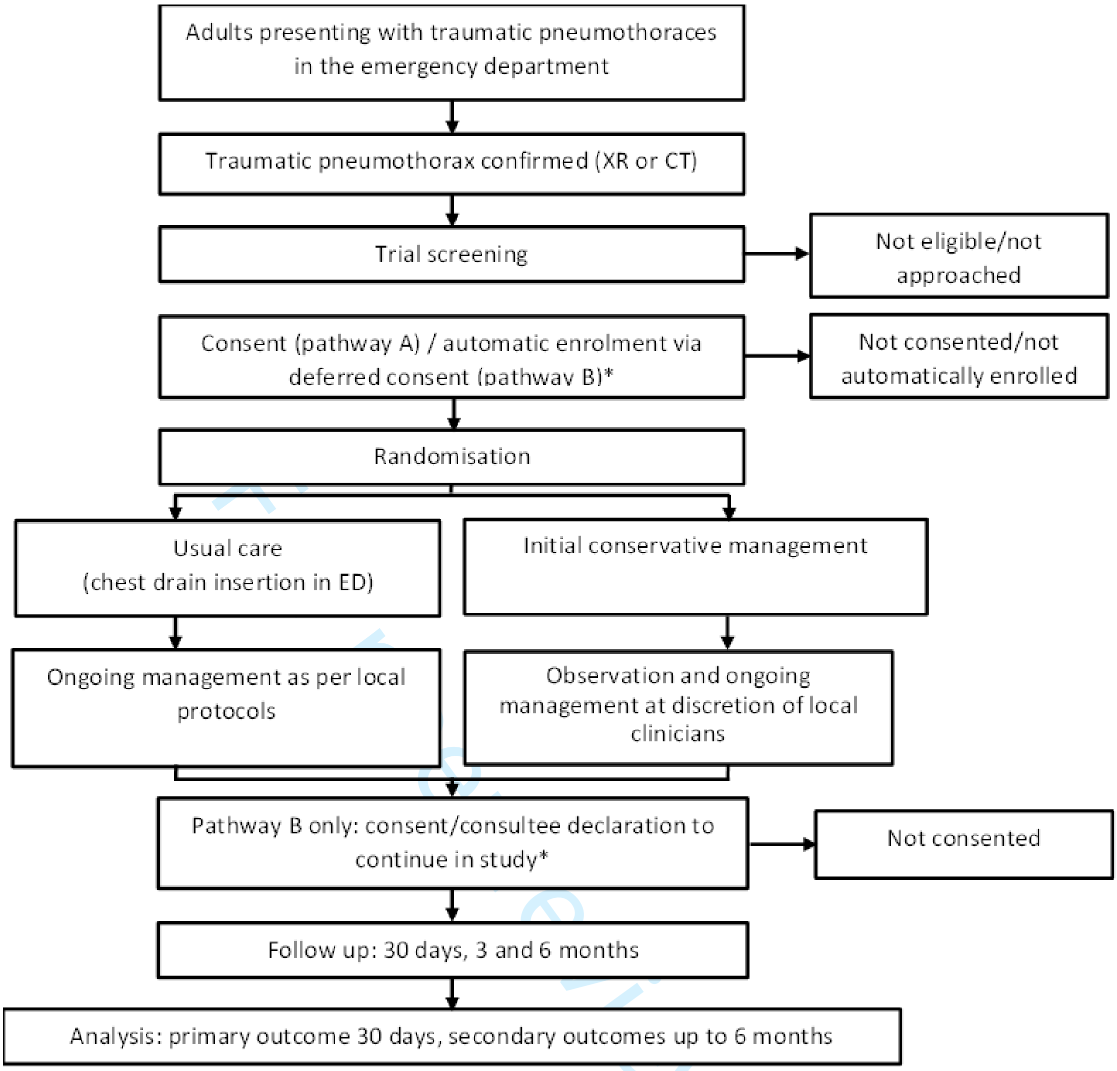

Figure 1. Trial schema illustrating the pathway for CoMiTED participants.

**It should be noted that patients without capacity are not being recruited in Scotland (Pathway B in England, Wales and Northern Ireland only).*

For peer review only

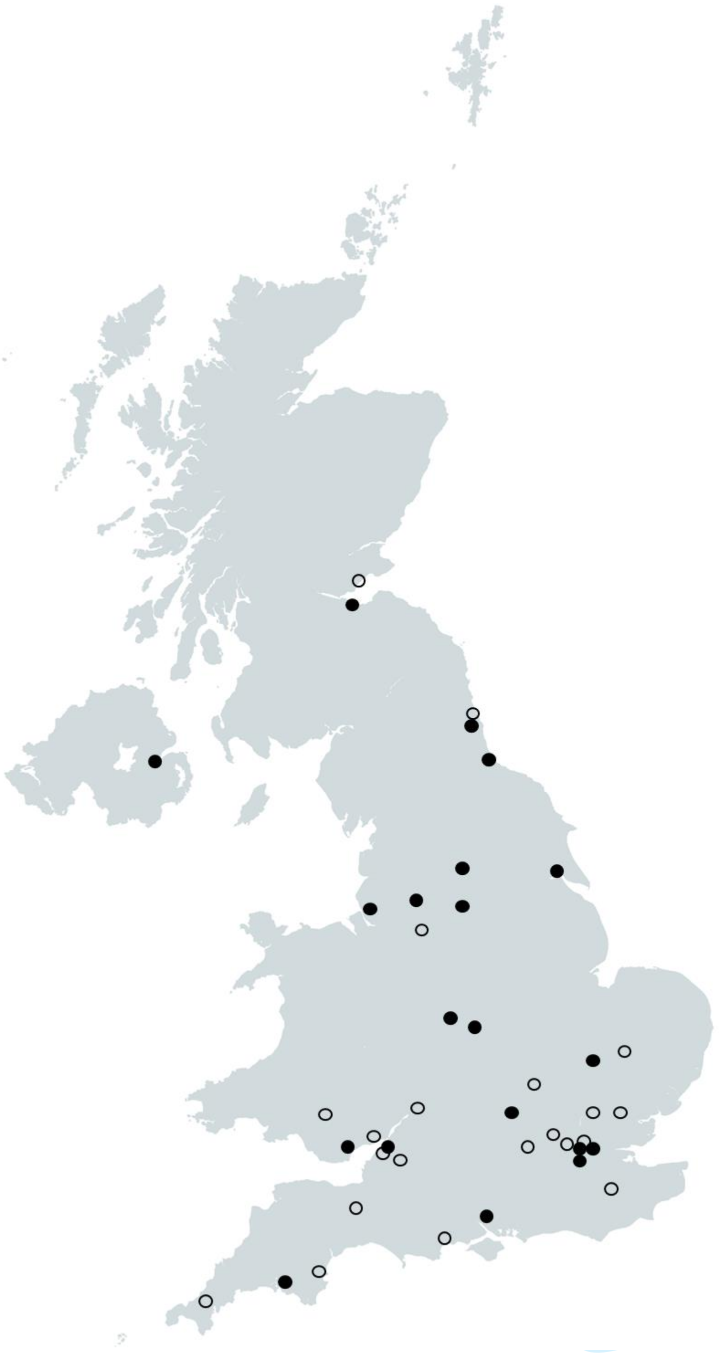

Figure 2. Distribution of participating organisations in the UK. Closed circles indicate major trauma centres and open circles indicate trauma units.

SPIRIT 2013 Checklist: Recommended items to address in a clinical trial protocol and related documents*

Section/item	ItemNo	Description	Addressed on page number
**Administrative information**			
Title	1	Descriptive title identifying the study design, population, interventions, and, if applicable, trial acronym	1
Trial registration	2a	Trial identifier and registry name. If not yet registered, name of intended registry	2
	2b	All items from the World Health Organization Trial Registration Data Set	N/A
Protocol version	3	Date and version identifier	9
Funding	4	Sources and types of financial, material, and other support	12
Roles and responsibilities	5a	Names, affiliations, and roles of protocol contributors	1, 12
	5b	Name and contact information for the trial sponsor	9
	5c	Role of study sponsor and funders, if any, in study design; collection, management, analysis, and interpretation of data; writing of the report; and the decision to submit the report for publication, including whether they will have ultimate authority over any of these activities	8, 9
	5d	Composition, roles, and responsibilities of the coordinating centre, steering committee, endpoint adjudication committee, data management team, and other individuals or groups overseeing the trial, if applicable (see Item 21a for data monitoring committee)	4, 8, 9

For peer review only - http://bmjopen.bmj.com/site/about/guidelines.xhtml

Introduction

Background and rationale	6a	Description of research question and justification for undertaking the trial, including summary of relevant studies (published and unpublished) examining benefits and harms for each intervention	2, 3
	6b	Explanation for choice of comparators	2, 3
Objectives	7	Specific objectives or hypotheses	3
Trial design	8	Description of trial design including type of trial (eg, parallel group, crossover, factorial, single group), allocation ratio, and framework (eg, superiority, equivalence, noninferiority, exploratory)	3

Methods: Participants, interventions, and outcomes

Study setting	9	Description of study settings (eg, community clinic, academic hospital) and list of countries where data will be collected. Reference to where list of study sites can be obtained	3, Figure 2
Eligibility criteria	10	Inclusion and exclusion criteria for participants. If applicable, eligibility criteria for study centres and individuals who will perform the interventions (eg, surgeons, psychotherapists)	Table 1
Interventions	11a	Interventions for each group with sufficient detail to allow replication, including how and when they will be administered	6
	11b	Criteria for discontinuing or modifying allocated interventions for a given trial participant (eg, drug dose change in response to harms, participant request, or improving/worsening disease)	6
	11c	Strategies to improve adherence to intervention protocols, and any procedures for monitoring adherence (eg, drug tablet return, laboratory tests)	6
	11d	Relevant concomitant care and interventions that are permitted or prohibited during the trial	6

For peer review only - http://bmjopen.bmj.com/site/about/guidelines.xhtml

Outcomes	12	Primary, secondary, and other outcomes, including the specific measurement variable (eg, systolic blood pressure), analysis metric (eg, change from baseline, final value, time to event), method of aggregation (eg, median, proportion), and time point for each outcome. Explanation of the clinical relevance of chosen efficacy and harm outcomes is strongly recommended	4, 5
Participant timeline	13	Time schedule of enrolment, interventions (including any run-ins and washouts), assessments, and visits for participants. A schematic diagram is highly recommended (see Figure)	Figure 1
Sample size	14	Estimated number of participants needed to achieve study objectives and how it was determined, including clinical and statistical assumptions supporting any sample size calculations	5
Recruitment	15	Strategies for achieving adequate participant enrolment to reach target sample size	5
**Methods: Assignment of interventions (for controlled trials)**			
Allocation:			
Sequence generation	16a	Method of generating the allocation sequence (eg, computer-generated random numbers), and list of any factors for stratification. To reduce predictability of a random sequence, details of any planned restriction (eg, blocking) should be provided in a separate document that is unavailable to those who enrol participants or assign interventions	8
Allocation concealment mechanism	16b	Mechanism of implementing the allocation sequence (eg, central telephone; sequentially numbered, opaque, sealed envelopes), describing any steps to conceal the sequence until interventions are assigned	6
Implementation	16c	Who will generate the allocation sequence, who will enrol participants, and who will assign participants to interventions	6
Blinding (masking)	17a	Who will be blinded after assignment to interventions (eg, trial participants, care providers, outcome assessors, data analysts), and how	2, 4

For peer review only - http://bmjopen.bmj.com/site/about/guidelines.xhtml

	17b	If blinded, circumstances under which unblinding is permissible, and procedure for revealing a participant's allocated intervention during the trial	N/A
**Methods: Data collection, management, and analysis**			
Data collection methods	18a	Plans for assessment and collection of outcome, baseline, and other trial data, including any related processes to promote data quality (eg, duplicate measurements, training of assessors) and a description of study instruments (eg, questionnaires, laboratory tests) along with their reliability and validity, if known. Reference to where data collection forms can be found, if not in the protocol	4, 5, 8, Table 2, Figure 1
	18b	Plans to promote participant retention and complete follow-up, including list of any outcome data to be collected for participants who discontinue or deviate from intervention protocols	6, 7
Data management	19	Plans for data entry, coding, security, and storage, including any related processes to promote data quality (eg, double data entry; range checks for data values). Reference to where details of data management procedures can be found, if not in the protocol	6
Statistical methods	20a	Statistical methods for analysing primary and secondary outcomes. Reference to where other details of the statistical analysis plan can be found, if not in the protocol	7, 8
	20b	Methods for any additional analyses (eg, subgroup and adjusted analyses)	7
	20c	Definition of analysis population relating to protocol non-adherence (eg, as randomised analysis), and any statistical methods to handle missing data (eg, multiple imputation)	7
**Methods: Monitoring**			
Data monitoring	21a	Composition of data monitoring committee (DMC); summary of its role and reporting structure; statement of whether it is independent from the sponsor and competing interests; and reference to where further details about its charter can be found, if not in the protocol. Alternatively, an explanation of why a DMC is not needed	9

	21b	Description of any interim analyses and stopping guidelines, including who will have access to these interim results and make the final decision to terminate the trial	N/A
Harms	22	Plans for collecting, assessing, reporting, and managing solicited and spontaneously reported adverse events and other unintended effects of trial interventions or trial conduct	8, 9
Auditing	23	Frequency and procedures for auditing trial conduct, if any, and whether the process will be independent from investigators and the sponsor	N/A
**Ethics and dissemination**			
Research ethics approval	24	Plans for seeking research ethics committee/institutional review board (REC/IRB) approval	11
Protocol amendments	25	Plans for communicating important protocol modifications (eg, changes to eligibility criteria, outcomes, analyses) to relevant parties (eg, investigators, REC/IRBs, trial participants, trial registries, journals, regulators)	11
Consent or assent	26a	Who will obtain informed consent or assent from potential trial participants or authorised surrogates, and how (see Item 32)	5, 6
	26b	Additional consent provisions for collection and use of participant data and biological specimens in ancillary studies, if applicable	N/A
Confidentiality	27	How personal information about potential and enrolled participants will be collected, shared, and maintained in order to protect confidentiality before, during, and after the trial	6, 8
Declaration of interests	28	Financial and other competing interests for principal investigators for the overall trial and each study site	12
Access to data	29	Statement of who will have access to the final trial dataset, and disclosure of contractual agreements that limit such access for investigators	11

For peer review only

Ancillary and post-trial care	30	Provisions, if any, for ancillary and post-trial care, and for compensation to those who suffer harm from trial participation	N/A
Dissemination policy	31a	Plans for investigators and sponsor to communicate trial results to participants, healthcare professionals, the public, and other relevant groups (eg, via publication, reporting in results databases, or other data sharing arrangements), including any publication restrictions	11
	31b	Authorship eligibility guidelines and any intended use of professional writers	N/A
	31c	Plans, if any, for granting public access to the full protocol, participant-level dataset, and statistical code	11
**Appendices**			
Informed consent materials	32	Model consent form and other related documentation given to participants and authorised surrogates	Upon request comited-trial@bristol.ac.uk
Biological specimens	33	Plans for collection, laboratory evaluation, and storage of biological specimens for genetic or molecular analysis in the current trial and for future use in ancillary studies, if applicable	N/A

*It is strongly recommended that this checklist be read in conjunction with the SPIRIT 2013 Explanation & Elaboration for important clarification on the items. Amendments to the protocol should be tracked and dated. The SPIRIT checklist is copyrighted by the SPIRIT Group under the Creative Commons "Attribution-NonCommercial-NoDerivs 3.0 Unported" license.

For peer review only

BMJ Open

Conservative management versus invasive management of significant traumatic pneumothoraces in the Emergency Department (The CoMiTED Trial): a study protocol for a randomised non-inferiority trial

Journal:	*BMJ Open*
Manuscript ID	bmjopen-2024-087464.R1
Article Type:	Protocol
Date Submitted by the Author:	01-May-2024
Complete List of Authors:	Blythe, Nicola; University of Bristol, Bristol Trials Centre<br>Coates, Katherine; North Bristol NHS Trust<br>Benger, Jonathan; University Hospitals Bristol and Weston NHS Foundation Trust; University of the West of England<br>Annaw, Ammar; University of Bristol, Bristol Trials Centre<br>Banks, Jonathan; University of Bristol, Bristol Trials Centre<br>Clement, Clare; University of the West of England<br>Clout, Madeleine; University of Bristol, Bristol Trials Centre<br>Edwards, Antoinette; The University of Manchester<br>Gaunt, Daisy; University of Bristol, Bristol Trials Centre<br>Kandiyali, Rebecca; University of Warwick<br>Lane, Athene; University of Bristol, Bristol Trials Centre<br>Lecky, Fiona; The University of Sheffield; Salford Royal NHS Trust, Emergency Department<br>Maskell, Nick; University of Bristol, Academic Respiratory Unit, School of Clinical Sciences; North Bristol NHS Trust, Respiratory Research Unit<br>Metcalfe, Chris; University of Bristol, Bristol Trials Centre<br>Platt, Marie; University of Bristol, Bristol Trials Centre<br>Rees, Sophie; University of Bristol, Bristol Trials Centre<br>Taylor, Jodi; University of Bristol, Bristol Trials Centre<br>Thompson, Julian; North Bristol NHS Trust<br>Walker, Steven; University of Bristol Academic Respiratory Unit; North Bristol NHS Trust<br>West, Douglas; University Hospitals Bristol and Weston NHS Foundation Trust<br>Carlton, Edward; North Bristol NHS Trust, Emergency Department, Southmead Hospital; University of Bristol
**Primary Subject Heading**:	Emergency medicine
Secondary Subject Heading:	Intensive care
Keywords:	ACCIDENT & EMERGENCY MEDICINE, Randomized Controlled Trial, TRAUMA MANAGEMENT

SCHOLARONE™
Manuscripts

Conservative management versus invasive management of significant traumatic pneumothoraces in the Emergency Department (The CoMiTED Trial): a study protocol for a randomised non-inferiority trial

Nicola M Blythe, Katherine Coates, Jonathan Benger, Ammar Annaw, Jonathan Banks, Clare Clement, Madeleine Clout, Antoinette Edwards, Daisy Gaunt, Rebecca Kandiyali, J Athene Lane, Fiona Lecky, Nick Maskell, Chris Metcalfe, Marie Platt, Sophie Rees, Jodi Taylor, Julian Thompson, Steven Walker, Douglas West and Edward Carlton.

Word count: 4122 (excluding title page, abstract, figures and tables, acknowledgements, contributions and references)

Number of tables: 2

Number of figures: 2 (submitted separately)

Corresponding author: Professor Edward Carlton, Emergency Department, Southmead Hospital, North Bristol NHS Trust, Southmead Road, Bristol, BS10 5NB. Email: ed.carlton@bristol.ac.uk.

Key words: accident and emergency medicine, trauma management, randomised controlled trial, traumatic pneumothorax, conservative management

For peer review only

ABSTRACT

Introduction

Traumatic pneumothoraces are present in 1 in 5 victims of severe trauma. Current guidelines advise chest drain insertion for most traumatic pneumothoraces, although very small pneumothoraces can be managed with observation at the treating clinician's discretion. There remains a large proportion of patients in whom there is clinical uncertainty as to whether an immediate chest drain is required, with no robust evidence to inform practice. Chest drains carry a high risk of complications such as bleeding and infection. The default to invasive treatment may be causing potentially avoidable pain, distress and complications. We are evaluating the clinical and cost-effectiveness of an initial conservative approach to the management of patients with traumatic pneumothoraces.

Methods and analysis

The CoMiTED trial is a multicentre, pragmatic parallel group, individually randomised controlled non-inferiority trial to establish whether initial conservative management of significant traumatic pneumothoraces is non-inferior to invasive management in terms of subsequent emergency pleural interventions, complications, pain, breathlessness, and quality of life. We aim to recruit 750 patients from at least 40 UK NHS hospitals. Patients allocated to the control (invasive management) group will have a chest drain inserted in the emergency department. For those in the intervention (initial conservative management) group, the treating clinician will be advised to manage the participant without chest drain insertion and undertake observation. The primary outcome is a binary measure of the need for one or more subsequent emergency pleural interventions within 30 days of randomisation. Secondary outcomes include complications, cost-effectiveness, patient-reported quality of life and patient and clinician views of the two treatment options; participants are followed up for 6 months.

Ethics and dissemination

For peer review only - http://bmjopen.bmj.com/site/about/guidelines.xhtml

This trial received approval from Wales Research Ethics Committee 4 (reference: 22/WA/0118) and the Health Research Authority. Results will be submitted for publication in a peer-reviewed journal.

Trial registration number ISRCTN35574247.

Strengths and Limitations of this study

- This is a pragmatic trial; once the initial decision has been made and patients have been allocated a treatment arm, all subsequent care and interventions are at the discretion of treating clinical teams.
- Patients will be recruited from the whole of the trauma spectrum to ensure results can be generalisable across the diverse trauma population.
- The trial involves economic evaluation to determine the clinical and cost effectiveness of initial conservative management versus invasive management of traumatic pneumothoraces.
- The trial has an integrated qualitative study in order to assess the acceptability of initial conservative management to patients and clinicians.
- Blinding to treatment allocation is not possible for clinicians or participants; only clinicians adjudicating primary outcome and researchers evaluating outcomes for the analyses will be blinded to treatment group.

For peer review only

INTRODUCTION

Injury is a leading cause of death among adults aged <45 years [1]. Traumatic pneumothoraces are present in 1 in 5 victims of severe trauma [2, 3]. We estimate from prior observational and survey work [4, 5] that around half of patients admitted to hospital with traumatic pneumothoraces will be treated with the insertion of a chest drain. Current guidelines advise chest drain insertion for most traumatic pneumothoraces, although very small pneumothoraces can be managed with observation at the treating clinician's discretion [6, 7]. For some patients with very large pneumothoraces, chest drain placement can reduce the risk of cardiorespiratory compromise [8]. However, there remains a large proportion of patients in whom there is clinical uncertainty as to whether an immediate chest drain is required [4]. Chest drains carry a high-risk of complications, such as bleeding and infection, in 15-30% of patients [9]. There is a lack of robust evidence to inform practice, and the default to invasive treatment may cause potentially avoidable patient harm.

In an analysis of >600 patients with traumatic pneumothoraces from 2012 to 2016, obtained from Trauma Audit & Research Network (TARN) data, 90% of patients treated without a chest drain did not require subsequent intervention [5], suggesting a potential role for conservative management. However, in this analysis, 50% of patients were initially treated with a chest drain and there was considerable clinical variation in those selected for this invasive procedure. In a 2020 international survey of 222 emergency physicians [4], utilising clinical vignettes of larger traumatic pneumothoraces, over 60% of clinicians would elect to insert a chest drain in the Emergency Department (ED), even without clinical compromise. Therefore, based on the observational studies and lack of robust data, we designed a randomised controlled trial (RCT) to assess the clinical and cost-effectiveness of an initial conservative approach to the management of patients with traumatic pneumothoraces. If we demonstrate that this approach achieves similar clinical outcomes, it will reduce the use of a painful, invasive and potentially harmful management strategy.

Prior to the start of the trial, we searched Medline for systematic reviews, and Medline, Embase, Cochrane Central, ClincalTrials.gov and the World Health Organisation (WHO) trials registry for trials

For peer review only - http://bmjopen.bmj.com/site/about/guidelines.xhtml

published. One systematic review from 2010 evaluated three small (total n=101) RCTs examining the safety of conservative management in small traumatic pneumothoraces [8]. This review suggested that conservative management may be at least as safe and effective as chest drain insertion. A further multicentre RCT of small pneumothoraces in severely injured patients in Canada concluded in 2021 [10]. These patients (142 in total) were all receiving positive pressure ventilation and current guidelines suggest chest drain insertion in all patients undergoing ventilation [2, 4]. The results showed no difference in mortality or intensive care unit (ICU) or hospital length of stay between patients who were conservatively managed and those who had chest drains inserted. The authors concluded that small traumatic pneumothoraces may be safely observed in patients undergoing ventilation and that the complications of chest drains remain unacceptably high. By including only patients undergoing ventilation (which is around 30% of the traumatic pneumothorax population in the UK [5]), the Canadian study did not fully address conservative management in the broader trauma population, as we are in this trial.

Aims and objectives

The Conservative Management in Traumatic Pneumothoraces in the Emergency Department (CoMiTED) trial will test whether initial conservative management of significant traumatic pneumothoraces is non-inferior to invasive management in terms of subsequent emergency pleural interventions, complications, pain, breathlessness, and quality of life.

Specific objectives are:

a) To establish if initial conservative management is non-inferior to invasive management regarding subsequent emergency pleural intervention over 30 days (or until death if sooner).

b) To determine whether conservative management improves health-related quality of life and other patient reported outcomes.

c) To determine the clinical and cost effectiveness of initial conservative management versus invasive management of traumatic pneumothoraces by measuring resource use, mortality and costs over the six months following injury.

d) To assess acceptability of initial conservative management to patients and clinicians.

METHODS AND ANALYSIS

Trial design

The CoMiTED trial is a pragmatic multicentre, parallel group, individually randomised controlled non-inferiority trial with an economic evaluation and integrated qualitative study.

Setting

The trial will recruit patients from approximately 40 NHS Major Trauma Centres and Trauma Units across the UK.

For peer review only - http://bmjopen.bmj.com/site/about/guidelines.xhtml

Trial population

Inclusion and exclusion are detailed in Table 1.

Inclusion Criteria	Exclusion Criteria
Presenting with traumatic pneumothorax/pneumothoraces	Treating clinician(s) believes injuries are incompatible with life
(Believed to be) 16 years and over	Patient in respiratory arrest
Treating clinician(s) believes either a chest drain or conservative management is a suitable initial treatment option	Haemothorax (associated with pneumothorax) requiring a chest drain in the opinion of the treating clinician(s)*
	Clinical or imaging evidence of tension pneumothorax in either lung at the point of randomisation
	Prisoner

Table 1. Table illustrating inclusion and exclusion criteria. Special Circumstances: In patients presenting with bilateral chest injury, if one lung of the patient qualifies, the patient can be enrolled, providing no exclusion criteria are met. Treatment of the eligible side follows the randomisation assignment, with the other side treated according to usual practice. If both sides qualify, both sides receive treatment according to the randomisation assignment. Patients who have received pre-hospital thoracostomies may still be enrolled, provided they fulfil the eligibility criteria. Where a participant who has received a prehospital thoracostomy is randomised to conservative management, local practice should be followed.

**Patients with an associated haemothorax are excluded due to this being a predictor of failure of conservative management [5].*

Primary outcome

The primary outcome is a binary measure of the need for one or more subsequent emergency pleural interventions in the eligible lung(s), from the point of randomisation up to 30 days. This excludes chest drain insertion in the ED for those allocated to the chest drain (control) group.

Reasons for subsequent emergency chest drain insertion may include (but are not limited to): clinically significant symptoms persisting despite adequate analgesia; chest pain or breathlessness preventing activity; a patient is unwilling to continue with conservative treatment; the patient's condition becomes physiologically unstable presumed secondary to pneumothorax; repeat chest radiograph shows an enlarging pneumothorax with physiological instability. Reasons for subsequent emergency pleural intervention are determined by local practice and recorded but are not controlled.

Whether a subsequent pleural intervention is deemed to be an emergency is adjudicated by a panel made up of independent expert clinicians from relevant specialties. The clinicians are blinded to allocation and presented with clinical and imaging vignettes of what happened to each participant and subsequently asked to determine whether, in their opinion, any subsequent pleural intervention that occurred within 30 days of randomisation was required due to an emergency. Consensus agreement is obtained by two members of the panel.

For peer review only - http://bmjopen.bmj.com/site/about/guidelines.xhtml

Secondary outcomes

The secondary outcomes will capture any differences between the allocated groups in terms of reduced pain, complications and improved health-related quality of life in the short to medium term, as well as inform a formal cost-effectiveness analysis.

Secondary outcomes are as follows: (i) All pleural interventions (including chest drain insertion in the ED) up to 30 days, (ii) All complications of pleural intervention up to 30 days, (iii) Total days of pleural drainage up to 30 days, (iv) Patient-reported pain [11], function and breathlessness [12] at baseline, 30 days, 3 and 6 months, (v) Quality of life [13, 14] at baseline, 30 days, 3 and 6 months, (vi) Total length of stay (hospital, critical care (including HDU) admission and readmission) up to 30 days, (vii) Adjudicated mortality at 30 days (pneumothorax or chest injury related), (viii) All-cause mortality at 6 months, (iv) Cost per quality-adjusted life year (QALY) at 6 months, (x) Patient views and experiences of conservative management/chest drain and (xi) Clinician views of conservative management/chest drain. For this trial, baseline patient reported outcome measures (PROMs) can be collected from as soon as feasible following randomisation and after treatment delivery (where appropriate) up to 7 days post-randomisation.

Sample size

We aim to recruit 750 participants and conduct approximately 25 patient interviews for the integrated qualitative study over 37 months (August 2022 – September 2025).

Observational data suggests that 10% of our trial population will require emergency pleural intervention following conservative management [5]. Our group recently identified a reintervention rate of 10% following initial chest drain insertion in a single UK site [5] and therefore anticipate the incidence of the primary outcome in the control group to be at least 10%.

Our patient and public involvement (PPI) contributors unanimously support the potential advantages of initial conservative management, such as avoiding an invasive procedure, improved mobilisation after injury, and reduced longer term pain. However, they also recognise the need to balance these benefits against the risk of avoidable harm. When asked, our PPI group felt that an increase of 5-10% in subsequent emergency pleural intervention would be acceptable compared to usual care, given the anticipated reduction in the overall number of chest drains in the intervention group. These views have been used to select a non-inferiority margin of 7.5%. We will conclude that the trial population can be safely managed conservatively if the incidence of subsequent emergency pleural intervention is no more than 7.5% higher in the intervention group than in the control group. If the incidence of the primary outcome is 10% in both groups, a sample size of 674 (337 in each group) will allow non-inferiority to be concluded with 90% power, when comparing a one-sided 97.5% confidence interval, for the absolute difference in primary outcome incidence, to a non-inferiority margin of 7.5%. Allowing 10% loss to follow-up increases the total sample size to 750.

Patient approach, recruitment and randomisation

Following eligibility assessment, eligible patients undergo a capacity assessment. If the patient has capacity, they are approached in the ED for their consent to take part. Where patients are judged to be unable to provide informed consent for themselves, then patients can be automatically enrolled under the waiver of consent (in countries where the waiver of consent is permitted for non-CTIMP trials). If patients regain capacity within 7 days post-randomisation, they are approached and asked whether they wish to provide consent to continue in the trial. If patients do not regain capacity

For peer review only - http://bmjopen.bmj.com/site/about/guidelines.xhtml

within 7 days of randomisation, a member of the research team seeks advice from a Personal Consultee or, if unavailable, a Nominated Consultee. The participant pathway is shown in Figure 1.

Patients are randomised in the ED immediately after traumatic pneumothorax has been diagnosed and consent provided / waiver of consent applied. Participants are allocated in a 1:1 ratio to either "chest drain insertion in the ED (control group)" or "initial conservative management (intervention group)" using a secure web-based randomisation system (Sealed Envelope, https://www.sealedenvelope.com/). Randomised allocation is minimised by three factors: 'trial site', 'currently ventilated' and 'penetrating injury'.

Trial intervention

In the intervention (initial conservative management) group, the treating clinician is advised to manage the participant without chest drain insertion and undertake observation and admission to a hospital ward or ICU. Given the pragmatic nature of this trial, all subsequent interventions and further imaging to evaluate pneumothorax resolution after the point of randomisation, is at the discretion of the treating clinical teams.

In the control group (chest drain insertion in the ED), the treating clinician is advised to insert a chest drain. Specific details of the procedure (including anaesthesia and insertion technique) is at the discretion of treating clinicians but will be recorded for trial purposes.

Data collection

Trial data is collected at baseline, 30 days, 3 months and 6 months and recorded by participating site team members onto case report forms (CRFs) and participant-completed questionnaires. Table 2 depicts the key assessments/outcome measures and participant-related procedures scheduled at various trial timepoints. These are entered into a REDCap database [15] for data cleaning and analysis. Access to the database is via a secure password-protected web interface.

**Data collection timepoint (→)**	**In the Emergency Department (ED)**		**Post-Randomisation Follow Ups**		
**Data capture / key trial procedure (↓)**	**Recruitment (Day 0)**	**Post-Recruitment (Baseline)**	**30 days**	**3 months**	**6 months**
Screening, Consent & Randomisation	X				
Case Report Form including safety reporting (CRF)	X	X	X	X	X
Sociodemographic Details		X			
Injury Details		X			
Injury Severity Score		X			
Comorbidities		X			

For peer review only - http://bmjopen.bmj.com/site/about/guidelines.xhtml

National Early Warning Score (routinely collected)		X			
PROMS; Pain (Brief Pain Inventory)		X	X	X	X
PROMS; Function (Brief Pain Inventory)		X	X	X	X
PROMS; Breathlessness (MRC dyspnoea scale)		X	X	X	X
PROMS; Quality of life (EQ-5D-5L)		X	X	X	X
PROMS; Impact Events Scale (IES-R)			X	X	X
Patient completed resource use questionnaire				X	X
Pleural interventions			X		
Complications			X		
Days of pleural drainage			X		
Length of stay (hospital and critical care (including HDU) admission, and readmission)			X		
Details of re-attendances to A&E or unplanned re-admissions within 30 days			X		
Mortality			X		X
Qualitative interviews			X		X

Table 2. Schedule of essential data capture and participant-related procedures.

For peer review only

Statistical analysis

Data obtained will be analysed according to the intention to treat principle, such that each participant's data will contribute to the findings for the group they were allocated to, irrespective of any subsequent diagnostic information or the treatment actually received. Reporting of the trial methodology and results will be according to the CONSORT guidelines [16]. The analysis will be pre-specified in detail in a statistical analysis plan, which will be made publicly available prior to the trial team having access to the data. The findings for the primary outcome measure will be presented as an absolute difference in incidence between conservative management and control groups, with the limit of the one-sided 97.5% confidence interval, compared to the non-inferiority bound of an absolute difference of a 7.5% higher incidence of the primary outcome in the conservative

For peer review only - http://bmjopen.bmj.com/site/about/guidelines.xhtml

management group. The primary analysis will be based on the observed data, but the potential impact of any missing primary outcome measures on the trial conclusions will be investigated in sensitivity analyses. If non-inferiority is demonstrated, evidence from the risk difference, two-sided 95% confidence interval, and p-value, will be presented to allow inference about the superiority of conservative management compared to usual care.

Health economic analysis

A cost utility analysis with a maximal time horizon of 6 months (corresponding to the period of maximal follow-up for patient-reported pain, dyspnoea and mortality) will be undertaken, since this is the time period that clinicians and patient advisors advise us is long enough to capture all relevant effects. However, it is possible that we will see convergence of costs and outcomes within 30 days (which corresponds to the primary outcome), and, to explore this, we will report cost-effectiveness at both 30 days and 6 months.

The QALY will be derived by applying the cross-walk algorithm to the EQ-5D-5L [13] and combining information on survival.

Resource use data is being collected on all NHS and personal social services care resources for trial participants up to 6 months. A patient-reported resource use questionnaire (note that the patient questionnaire will incorporate ModRUM [17], with the addition of some trial-specific questions) at 3 and 6 months will provide additional data on primary and community care resource after discharge.

Utilising medical notes from patients coded for chest drain insertion at one site, a set of assumptions have been established detailing staff, equipment, analgesia and imaging use relating to chest drain insertion and other high-cost pleural interventions in different settings and these will be reviewed by clinicians at participating organisations for accuracy. The aim of this is to enhance our understanding of the trauma pathway and to inform and validate our costing approach.

Qualitative analysis

The qualitative research aims to provide a comprehensive and in-depth understanding of the acceptability of initial conservative management versus chest drain to patients and clinicians by conducting interviews using topic guides. Patients and consultees will be approached at least eight weeks after randomisation. Interviews will explore patient and consultee views and experiences of conservative management or chest drain insertion in the short to medium term, including impact on their daily life, positive and negative aspects of the treatment, pain, post-procedure recovery, subsequent treatments and return to activities. To enhance the understanding of clinician acceptability of initial conservative treatment and its implementation in wider practice, we will also interview clinicians involved in the trial patient pathway. Interviews will explore views of initial conservative management/chest drain, potential hidden benefits of initial conservative management, barriers to and facilitators for introducing initial conservative management into practice and what influences decision-making concerning traumatic pneumothorax management.

Maximum variation/purposive sampling will be used when possible, with the aim of achieving diversity in terms of participant characteristics. Anonymised transcripts will be analysed using reflexive thematic analysis [18]. Transcripts will be coded for key categories and concepts, using deductive coding (based on the research aims and Theoretical Framework of Acceptability) [19] and inductive coding (developing new codes based on issues emerging from the data) with the aid of NVivo software. Findings will be considered in relation to quantitative results and provide enhanced understanding of chest injury management in the emergency context.

For peer review only - http://bmjopen.bmj.com/site/about/guidelines.xhtml

Safety

Participant safety will be monitored by the Trial Management Group (TMG), Sponsor and oversight committees (Trial Steering Committee and Data Monitoring Committee). The protocol contains a list of events that can be expected in this patient population. If an expected serious adverse event (SAE) is prolonged or more serious than expected, this will be reported as an unexpected SAE.

All SAEs, expected non-serious adverse events (AEs) and non-serious AEs caused by pleural interventions (which occur up to 30 days post-randomisation for the latter) will be recorded in CRFs and monitored. SAEs that are both related to the trial (i.e. resulted from conservative management or administration of a research procedure) and unexpected (i.e. not listed in the protocol as expected) are suspected unexpected serious adverse reactions (SUSARs) and will be subject to reporting to the Sponsor and Research Ethics Committee (REC). We do not expect any AEs or SAEs related to conservative management (above those expected of the control arm, i.e. standard care).

Patient and Public Involvement

A PPI group made up of PPI co-applicants/members, and supplemented through networking and outreach work, meet as needed throughout the duration of the trial to ensure an iterative and responsive PPI strategy. Our PPI group have fed into all aspects of trial design, provided feedback on trial documents and have been involved in maximising retention of participants. A group of knife crime and violence reduction professionals from a boxing group in Bristol, Empire Fighting Chance, meet separately to address this important element of the trial.

Trial management and oversight

The Chief Investigators take overall responsibility for managing the trial. Bristol Trials Centre, a UK Clinical Research Collaboration-registered trials unit, is responsible for the preparation of trial documents, site initiation visits and training, day-to-day running of the trial and monitoring of centres. The TMG oversees the trial and meets bimonthly to review progress. The trial steering committee meets biannually to review conduct and progress and the data monitoring committee at least annually to review data completion and safety. The trial Sponsor is North Bristol NHS Trust, who oversees the trial and has ultimate responsibility for any decision about its continuation.

Changes to trial protocol

Since the first trial protocol was approved by the Research and Ethics Committee (V2.0, dated 11 May 2022), there have been three amendments to the protocol (current version is version 5.0, dated 08 June 2023). The first amendment (protocol version 3.0, 07 July 2022) clarified the eligibility criteria where bilateral pneumothoraces are present. The second amendment (protocol version 4.0, 15 December 2022), amended the key inclusion criterion from '*in whom the treating clinician(s) are uncertain if a chest drain is required*' to '*in whom the treating clinician(s) believes either a chest drain or conservative management is a suitable initial treatment option*', based on feedback from participating organisations. The third amendment, (protocol version 5.0, 08 June 2023), allowed the recruitment of patients at NHS organisations in Scotland, via informed consent only, due to differences in legalities for patients judged not to have capacity in Scotland.

For peer review only - http://bmjopen.bmj.com/site/about/guidelines.xhtml

DISCUSSION

This trial investigating initial conservative management of traumatic pneumothoraces is a pragmatic multicentre RCT aiming to establish whether this approach is non-inferior to chest drain insertion in terms of clinical and cost-effectiveness. Should an initial conservative approach prove non-inferior to invasive management, this is likely to lead to widespread changes in practice and reduce avoidable harm from chest drain insertion.

We recognise that this trial is both methodologically complex and will be a challenge to deliver in an emergency setting. The following aspects have been considered in order to ensure the trial can be successfully delivered and answer the aims and objectives.

Clinical Equipoise

Equipoise is the key to our third inclusion criterion which relates to whether the treating clinician(s) would feel comfortable treating a patient's traumatic pneumothorax with a chest drain or conservative management. The subjective nature of this inclusion criterion has been our most significant challenge to date. During the initial stages of recruitment, this inclusion criterion read '*in whom the treating clinician(s) are uncertain if a chest drain is required*'. During the early stages of recruitment, both the trial team and the qualitative research team received feedback from site teams that clinicians may have been perceiving this as questioning their confidence in their clinical decision-making, rather than the intended 'research uncertainty', and that eligible patients may be being excluded due to this. An amendment was implemented to change this key inclusion criterion to '*in whom the treating clinician(s) believes either a chest drain or conservative management is a suitable initial treatment option*'. The trial team have emphasised when training site teams that a patient should be considered if the treating clinician acknowledges that the patient could be treated differently if seen by a colleague, or if they presented at a different NHS hospital. During training, case studies (anonymised radiology images) are shared with sites to illustrate the variation in the sizes of traumatic pneumothoraces within the trial, including mention of factors which affected decision-making (e.g. presence of surgical emphysema, ventilation status, body mass index).

In addition, variation in embedded practice within the specialty groups involved in decision-making has been noticeable throughout the duration of the trial. Generally speaking, emergency clinicians seem more comfortable with treating small to moderate traumatic pneumothoraces conservatively, whereas there has been some reluctance from the surgical community, with a preference for chest drain insertion often observed. This may be due to concerns about the potential increased need for chest drain insertion on hospital wards and the resource available to do this. Site teams have been reassured that the number of patients recruited at each site will be relatively small, that only half of the patients will be allocated to the conservative management arm and, in addition, the need for subsequent intervention is likely to be low at ~1 in 10, based on previous observational data [5].

Recruitment of multiply-injured patients

We anticipated that 40% of participants recruited would be intubated and ventilated, based on TARN data. However, in June 2023, analysis showed that only 8% of patients recruited were intubated and ventilated at the time of randomisation. This may have been due to a preference for invasive management in positively pressure ventilated patients, despite prior evidence demonstrating that ventilation does not predict failure of conservative management [5,10]. The TMG were concerned that this may affect the generalisability of the trial's results. The trial team

For peer review only - http://bmjopen.bmj.com/site/about/guidelines.xhtml

have been engaging the critical care community via infographics and webinars and have since seen an increase in the proportion of intubated and ventilated patients included to 15%.

Trial information provision

Due to the heterogenous target population, we have trial information material available in a variety of formats to facilitate maximal participation in eligible patients. We created a patient information video (https://comited.blogs.bristol.ac.uk/information-for-patients/) which was aimed towards younger people. Patients are able to provide consent to participate after watching the video, with a detailed patient information sheet also provided for further reading. Individuals from the charity Empire Fighting Chance made a valuable contribution towards creation of the patient information video, providing feedback and suggestions of ways to ensure the video was relevant to the target group. The video has received positive feedback from participating site research teams and has enabled at least one patient who was unable to read to participate in the trial.

There are two pathways via which to enrol a patient into the trial: obtaining informed consent from those with capacity or recruiting those lacking capacity (temporarily or permanently) under the emergency waiver of consent [20]. In some cases, patients may initially seem alert (e.g. be standing, walking or talking), and this may be mistaken for capacity to make an informed decision about participation in research, especially in those who are under the influence of alcohol or drugs (recreational or medication) or in extreme pain. We have found that, in some situations, such patients do not recall the 'informed consent' discussion when spoken to at a later date so we encourage site teams to keep this in mind and to enrol patients under the waiver of consent if they feel this is appropriate.

The CoMiTED research team, alongside collaborators, created a short video for those who were temporarily lacking capacity at the time they were admitted to the ED and enrolled under the waiver of consent (https://comited.blogs.bristol.ac.uk/for-patients-who-have-recovered-capacity/). The video explains that they were too unwell to be asked about taking part at the time a decision about their treatment needed to be made, therefore doctors decided that it was safe for the patient to take part. The video explains that, now that the patient is well enough, they are being approached with details of the trial and being asked if they wish to provide consent for continued participation. The video is not specific to the CoMiTED trial and is available for use in all emergency care trials utilising the waiver of consent.

Conclusion

The CoMiTED trial is a multicentre pragmatic RCT which has been designed to generate new evidence around the management of patients with traumatic pneumothoraces and has the potential to lead to significant changes in clinical practice.

Trial status

Recruitment to the trial began in August 2022, with an internal pilot to test feasibility. The trial is currently recruiting at 41 UK organisations (20 major trauma centres and 21 trauma units, distribution is shown in Figure 2); and, as of 10/04/2024, 235 participants have been recruited across 35 sites.

For peer review only - http://bmjopen.bmj.com/site/about/guidelines.xhtml

Ethics and dissemination

This trial was given a favourable opinion by Wales Research Ethics Committee 4 (reference: 22/WA/0118) and received approval from the Health Research Authority.

Trial participants are kept informed of trial progress via newsletters. A trial-specific X (previously Twitter) account, @CoMiTEDTrial, is used to promote the trial, provide updates and will disseminate findings. We aim to publish our primary, peer-reviewed manuscript in a high impact medical journal and present our findings at multiple conferences. We will communicate our findings to the British Thoracic Society, National Institute for Health and Care Excellence and NHS England to incorporate the work into relevant national guidelines. The dataset will be published in the publicly available University of Bristol Research Data repository https://www.bristol.ac.uk/staff/researchers/data/accessing-research-data/.

Acknowledgments

The authors thank all of the research and clinical team members at participating sites, members of the independent Trial Steering Committee, Data Monitoring Committee and adjudication panel, members of the patient and public involvement group and all of the participants who have taken part in this research so far. The authors would also like to thank Kate Beckett, Holly Mckeon and Amanda Lewis for their contributions.

Contributors

EC and JB are the Co-Chief Investigators, identified the funding opportunity and designed the trial, alongside co-applicants. NB set-up and manages the day-to-day running of the trial and assembled the manuscript from the trial protocol. KC and MP manage the day-to-day running of the trial. CM supervises the statistical analysis, and will write the statistical analysis plan. DG provides progress reports to the Trial Management Group and the independent oversight committees, and will complete the statistical analysis. SR leads the qualitative research, alongside JBa and, previously, CC. RK leads the health economics research, alongside AA. JT, AL & MC provide senior trial management oversight and advice. AE, FL, NM, SW, JTh and DW provide advice as part of the Trial Management Group. All authors were involved in preparation of the trial protocol and have read and approved the final manuscript.

Funding

The project is funded by the National Institute for Health and Care Research (NIHR) Health Technology Assessment Programme (HTA) (ref: NIHR132889). The views expressed are those of the authors and not necessarily those of the NIHR or the Department of Health and Social Care. This trial has been designed and delivered in collaboration with Bristol Trials Centre, a UK Clinical Research Collaboration-registered clinical trials unit, which is in receipt of NIHR clinical trials unit support funding.

Competing interests

The authors declare that they have no competing interests.

For peer review only - http://bmjopen.bmj.com/site/about/guidelines.xhtml

References

1. Moran C, Lecky F, Bouamra O, et al. Changing the System – Major Trauma Patients and Their Outcomes in the NHS (England) 2008–17. Lancet Eclinical Medicine. 2018;2:13-21.
2. Di Bartolomeo S, Sanson G, Nardi G, et al. A population-based study on pneumothorax in severely traumatized patients. J Trauma. 2001;51(4):677-82.
3. Kshettry VR, Bolman RM, 3rd. Chest trauma. Assessment, diagnosis, and management. Clin Chest Med. 1994;15(1):137-46.
4. Avery P, Watkins M, Benger J, et al. Current management of moderate to severe traumatic pneumothoraces: a survey of emergency clinicians. Emerg Med J. 2021.
5. Walker SP, Barratt SL, Thompson J, et al. Conservative Management in Traumatic Pneumothoraces: An Observational Study. Chest. 2018;153(4):946-53.
6. Surgeons Aco. Advanced Trauma Life Support® Student Course Manual. 10th Edition ed:Chicago: American College of Surgeons; 2018.
7. Thies KMG, P. European Trauma Course 4th edition. 2016.
8. Yadav K, Jalili M, Zehtabchi S. Management of traumatic occult pneumothorax. Resuscitation. 2010;81(9):1063-8.
9. Menger R, Telford G, Kim P, et al. Complications following thoracic trauma managed with tube thoracostomy. Injury. 2012;43(1):46-50.
10. Clements TW, Sirois M, Parry N, et al. OPTICC: A multicentre trial of Occult Pneumothoraces subjected to mechanical ventilation: The final report. American journal of surgery. 2021;221(6).
11. Tan G, Jensen MP, Thornby JI, at al. Validation of the Brief Pain Inventory for chronic nonmalignant pain. J Pain. 2004;5(2):133-7.
12. Stenton C. The MRC breathlessness scale. Occupational Medicine. 2008;58(3):226-7.
13. EuroQol G. EuroQol--a new facility for the measurement of health-related quality of life. Health Policy. 1990;16(3):199-208.
14. Weiss DS, Marmar C, Wilson JP, et al. Assessing psychological trauma and PTSD. 1997.
15. Harris PA, Taylor R, Thielke R, et al. Research electronic data capture (REDCap)–a metadata-driven methodology and workflow process for providing translational research informatics support. J Biomed Inform. 2009;42(2):377–81.
16. CONSORT Group. Consort transparent reporting of trials, 2010. Available: http://www. consort- statement.org/
17. Garfield K, Husbands S, Thorn JC, et al. Development of a brief, generic, modular resource-use measure (ModRUM): cognitive interviews with patients. BMC Health Services Research. 2021;21(1):1-12.
18. Braun V, Clarke V. Reflecting on reflexive thematic analysis. Qualitative Research in Sport, Exercise and Health. 2019;11(4):589-97.
19. Sekhon M, Cartwright M, Francis JJ. Acceptability of healthcare interventions: an overview of reviews and development of a theoretical framework. BMC Health Serv Res. 2017;17(1):88.
20. Health Research Authority (HRA). Principles of consent: Emergency Research (England and Wales) [19 November 2021]. Available from: http://www.hradecisiontools.org.uk/consent/principles-emergency-EnglandandWales.html.

For peer review only - http://bmjopen.bmj.com/site/about/guidelines.xhtml

Figure legends (figures submitted separately)

Figure 1. Trial schema illustrating the pathway for CoMiTED participants.

**It should be noted that patients without capacity are not being recruited in Scotland (Pathway B in England, Wales and Northern Ireland only).*

Figure 2. Distribution of participating organisations in the UK. Closed circles indicate major trauma centres and open circles indicate trauma units.

For peer review only

For peer review only - http://bmjopen.bmj.com/site/about/guidelines.xhtml

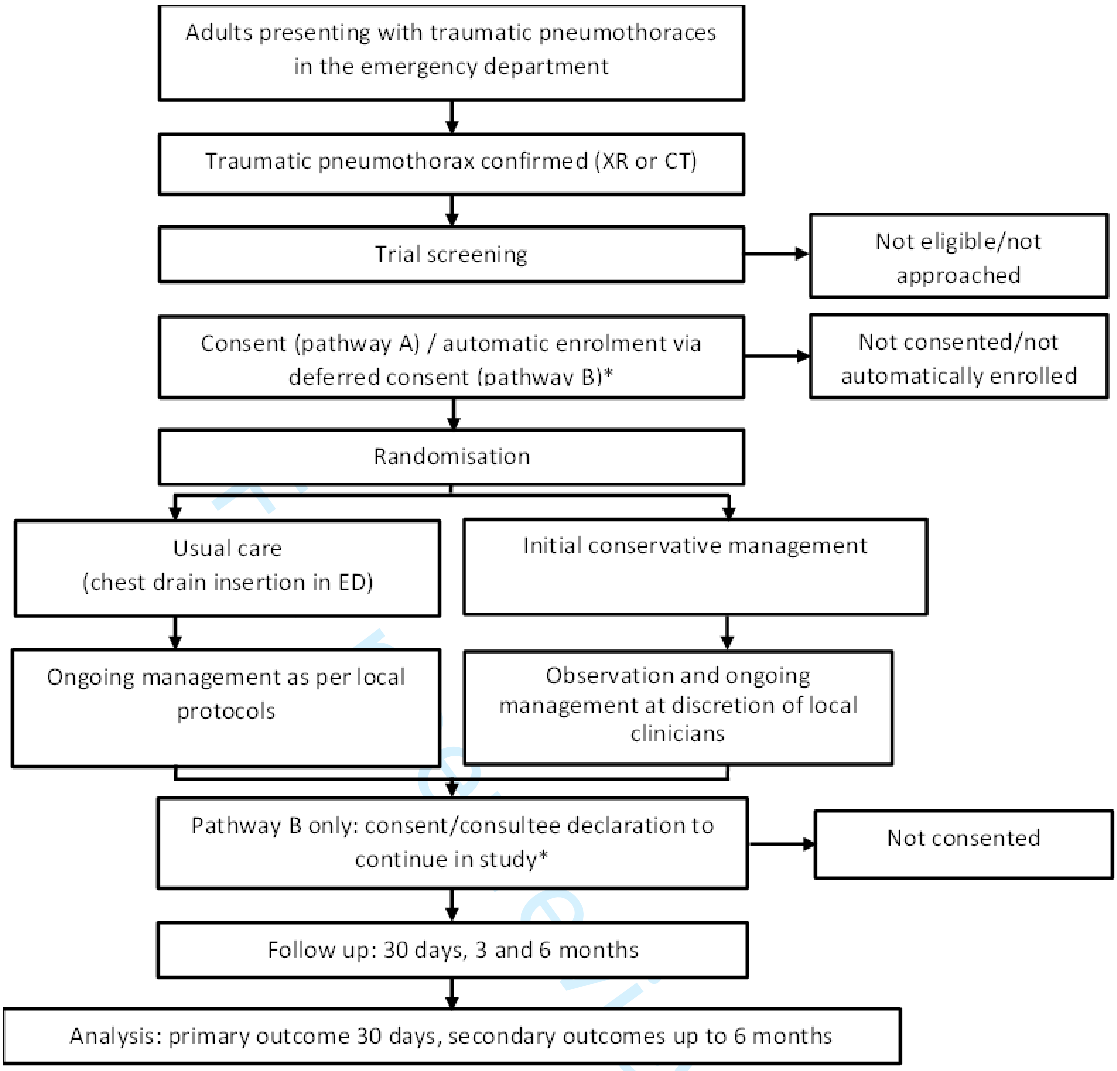

Figure 1. Trial schema illustrating the pathway for CoMiTED participants.

**It should be noted that patients without capacity are not being recruited in Scotland (Pathway B in England, Wales and Northern Ireland only).*

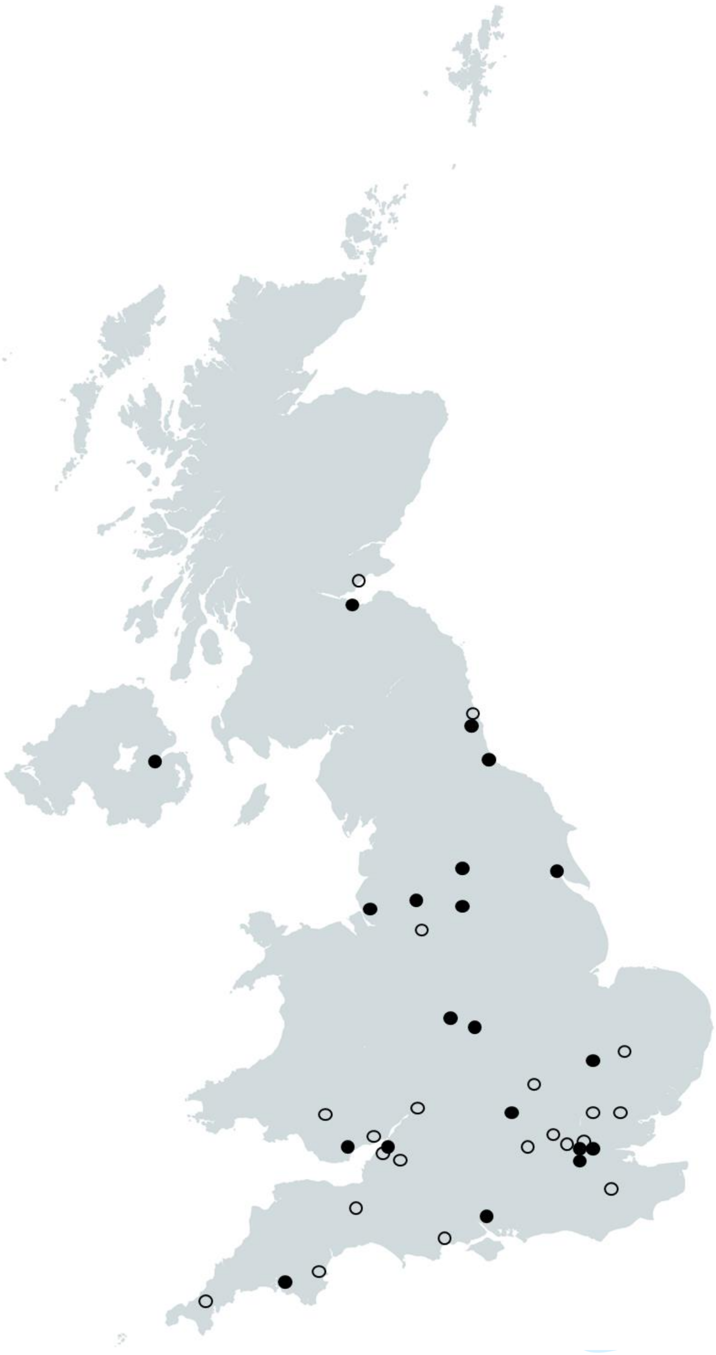

Figure 2. Distribution of participating organisations in the UK. Closed circles indicate major trauma centres and open circles indicate trauma units.

SPIRIT

STANDARD PROTOCOL ITEMS: RECOMMENDATIONS FOR INTERVENTIONAL TRIALS

SPIRIT 2013 Checklist: Recommended items to address in a clinical trial protocol and related documents*

**Section/item**	**ItemNo**	**Description**	**Addressed on page number**
**Administrative information**			
Title	1	Descriptive title identifying the study design, population, interventions, and, if applicable, trial acronym	1
Trial registration	2a	Trial identifier and registry name. If not yet registered, name of intended registry	2
	2b	All items from the World Health Organization Trial Registration Data Set	N/A
Protocol version	3	Date and version identifier	9
Funding	4	Sources and types of financial, material, and other support	12
Roles and responsibilities	5a	Names, affiliations, and roles of protocol contributors	1, 12
	5b	Name and contact information for the trial sponsor	9
	5c	Role of study sponsor and funders, if any, in study design; collection, management, analysis, and interpretation of data; writing of the report; and the decision to submit the report for publication, including whether they will have ultimate authority over any of these activities	8, 9
	5d	Composition, roles, and responsibilities of the coordinating centre, steering committee, endpoint adjudication committee, data management team, and other individuals or groups overseeing the trial, if applicable (see Item 21a for data monitoring committee)	4, 8, 9

For peer review only

**Introduction**			
Background and rationale	6a	Description of research question and justification for undertaking the trial, including summary of relevant studies (published and unpublished) examining benefits and harms for each intervention	2, 3
	6b	Explanation for choice of comparators	2, 3
Objectives	7	Specific objectives or hypotheses	3
Trial design	8	Description of trial design including type of trial (eg, parallel group, crossover, factorial, single group), allocation ratio, and framework (eg, superiority, equivalence, noninferiority, exploratory)	3
**Methods: Participants, interventions, and outcomes**			
Study setting	9	Description of study settings (eg, community clinic, academic hospital) and list of countries where data will be collected. Reference to where list of study sites can be obtained	3, Figure 2
Eligibility criteria	10	Inclusion and exclusion criteria for participants. If applicable, eligibility criteria for study centres and individuals who will perform the interventions (eg, surgeons, psychotherapists)	Table 1
Interventions	11a	Interventions for each group with sufficient detail to allow replication, including how and when they will be administered	6
	11b	Criteria for discontinuing or modifying allocated interventions for a given trial participant (eg, drug dose change in response to harms, participant request, or improving/worsening disease)	6
	11c	Strategies to improve adherence to intervention protocols, and any procedures for monitoring adherence (eg, drug tablet return, laboratory tests)	6
	11d	Relevant concomitant care and interventions that are permitted or prohibited during the trial	6

For peer review only - http://bmjopen.bmj.com/site/about/guidelines.xhtml

Outcomes	12	Primary, secondary, and other outcomes, including the specific measurement variable (eg, systolic blood pressure), analysis metric (eg, change from baseline, final value, time to event), method of aggregation (eg, median, proportion), and time point for each outcome. Explanation of the clinical relevance of chosen efficacy and harm outcomes is strongly recommended	4, 5
Participant timeline	13	Time schedule of enrolment, interventions (including any run-ins and washouts), assessments, and visits for participants. A schematic diagram is highly recommended (see Figure)	Figure 1
Sample size	14	Estimated number of participants needed to achieve study objectives and how it was determined, including clinical and statistical assumptions supporting any sample size calculations	5
Recruitment	15	Strategies for achieving adequate participant enrolment to reach target sample size	5
**Methods: Assignment of interventions (for controlled trials)**			
Allocation:			
Sequence generation	16a	Method of generating the allocation sequence (eg, computer-generated random numbers), and list of any factors for stratification. To reduce predictability of a random sequence, details of any planned restriction (eg, blocking) should be provided in a separate document that is unavailable to those who enrol participants or assign interventions	8
Allocation concealment mechanism	16b	Mechanism of implementing the allocation sequence (eg, central telephone; sequentially numbered, opaque, sealed envelopes), describing any steps to conceal the sequence until interventions are assigned	6
Implementation	16c	Who will generate the allocation sequence, who will enrol participants, and who will assign participants to interventions	6
Blinding (masking)	17a	Who will be blinded after assignment to interventions (eg, trial participants, care providers, outcome assessors, data analysts), and how	2, 4

	17b	If blinded, circumstances under which unblinding is permissible, and procedure for revealing a participant's allocated intervention during the trial	N/A
**Methods: Data collection, management, and analysis**			
Data collection methods	18a	Plans for assessment and collection of outcome, baseline, and other trial data, including any related processes to promote data quality (eg, duplicate measurements, training of assessors) and a description of study instruments (eg, questionnaires, laboratory tests) along with their reliability and validity, if known. Reference to where data collection forms can be found, if not in the protocol	4, 5, 8, Table 2, Figure 1
	18b	Plans to promote participant retention and complete follow-up, including list of any outcome data to be collected for participants who discontinue or deviate from intervention protocols	6, 7
Data management	19	Plans for data entry, coding, security, and storage, including any related processes to promote data quality (eg, double data entry; range checks for data values). Reference to where details of data management procedures can be found, if not in the protocol	6
Statistical methods	20a	Statistical methods for analysing primary and secondary outcomes. Reference to where other details of the statistical analysis plan can be found, if not in the protocol	7, 8
	20b	Methods for any additional analyses (eg, subgroup and adjusted analyses)	7
	20c	Definition of analysis population relating to protocol non-adherence (eg, as randomised analysis), and any statistical methods to handle missing data (eg, multiple imputation)	7
**Methods: Monitoring**			
Data monitoring	21a	Composition of data monitoring committee (DMC); summary of its role and reporting structure; statement of whether it is independent from the sponsor and competing interests; and reference to where further details about its charter can be found, if not in the protocol. Alternatively, an explanation of why a DMC is not needed	9

For peer review only - http://bmjopen.bmj.com/site/about/guidelines.xhtml

	21b	Description of any interim analyses and stopping guidelines, including who will have access to these interim results and make the final decision to terminate the trial	N/A
Harms	22	Plans for collecting, assessing, reporting, and managing solicited and spontaneously reported adverse events and other unintended effects of trial interventions or trial conduct	8, 9
Auditing	23	Frequency and procedures for auditing trial conduct, if any, and whether the process will be independent from investigators and the sponsor	N/A
**Ethics and dissemination**			
Research ethics approval	24	Plans for seeking research ethics committee/institutional review board (REC/IRB) approval	11
Protocol amendments	25	Plans for communicating important protocol modifications (eg, changes to eligibility criteria, outcomes, analyses) to relevant parties (eg, investigators, REC/IRBs, trial participants, trial registries, journals, regulators)	11
Consent or assent	26a	Who will obtain informed consent or assent from potential trial participants or authorised surrogates, and how (see Item 32)	5, 6
	26b	Additional consent provisions for collection and use of participant data and biological specimens in ancillary studies, if applicable	N/A
Confidentiality	27	How personal information about potential and enrolled participants will be collected, shared, and maintained in order to protect confidentiality before, during, and after the trial	6, 8
Declaration of interests	28	Financial and other competing interests for principal investigators for the overall trial and each study site	12
Access to data	29	Statement of who will have access to the final trial dataset, and disclosure of contractual agreements that limit such access for investigators	11

For peer review only

Ancillary and post-trial care	30	Provisions, if any, for ancillary and post-trial care, and for compensation to those who suffer harm from trial participation	N/A
Dissemination policy	31a	Plans for investigators and sponsor to communicate trial results to participants, healthcare professionals, the public, and other relevant groups (eg, via publication, reporting in results databases, or other data sharing arrangements), including any publication restrictions	11
	31b	Authorship eligibility guidelines and any intended use of professional writers	N/A
	31c	Plans, if any, for granting public access to the full protocol, participant-level dataset, and statistical code	11
**Appendices**			
Informed consent materials	32	Model consent form and other related documentation given to participants and authorised surrogates	Upon request comited-trial@bristol.ac.uk
Biological specimens	33	Plans for collection, laboratory evaluation, and storage of biological specimens for genetic or molecular analysis in the current trial and for future use in ancillary studies, if applicable	N/A

*It is strongly recommended that this checklist be read in conjunction with the SPIRIT 2013 Explanation & Elaboration for important clarification on the items. Amendments to the protocol should be tracked and dated. The SPIRIT checklist is copyrighted by the SPIRIT Group under the Creative Commons "Attribution-NonCommercial-NoDerivs 3.0 Unported" license.

BMJ Open

Conservative management versus invasive management of significant traumatic pneumothoraces in the Emergency Department (The CoMiTED Trial): a study protocol for a randomised non-inferiority trial

Journal:	*BMJ Open*
Manuscript ID	bmjopen-2024-087464.R2
Article Type:	Protocol
Date Submitted by the Author:	16-May-2024
Complete List of Authors:	Blythe, Nicola; University of Bristol, Bristol Trials Centre<br>Coates, Katherine; North Bristol NHS Trust<br>Benger, Jonathan; University Hospitals Bristol and Weston NHS Foundation Trust; University of the West of England<br>Annaw, Ammar; University of Bristol, Bristol Trials Centre<br>Banks, Jonathan; University of Bristol, Bristol Trials Centre<br>Clement, Clare; University of the West of England<br>Clout, Madeleine; University of Bristol, Bristol Trials Centre<br>Edwards, Antoinette; The University of Manchester<br>Gaunt, Daisy; University of Bristol, Bristol Trials Centre<br>Kandiyali, Rebecca; University of Warwick<br>Lane, Athene; University of Bristol, Bristol Trials Centre<br>Lecky, Fiona; The University of Sheffield; Salford Royal NHS Trust, Emergency Department<br>Maskell, Nick; University of Bristol, Academic Respiratory Unit, School of Clinical Sciences; North Bristol NHS Trust, Respiratory Research Unit<br>Metcalfe, Chris; University of Bristol, Bristol Trials Centre<br>Platt, Marie; University of Bristol, Bristol Trials Centre<br>Rees, Sophie; University of Bristol, Bristol Trials Centre<br>Taylor, Jodi; University of Bristol, Bristol Trials Centre<br>Thompson, Julian; North Bristol NHS Trust<br>Walker, Steven; University of Bristol Academic Respiratory Unit; North Bristol NHS Trust<br>West, Douglas; University Hospitals Bristol and Weston NHS Foundation Trust<br>Carlton, Edward; North Bristol NHS Trust, Emergency Department, Southmead Hospital; University of Bristol
**Primary Subject Heading**:	Emergency medicine
Secondary Subject Heading:	Intensive care
Keywords:	ACCIDENT & EMERGENCY MEDICINE, Randomized Controlled Trial, TRAUMA MANAGEMENT

Conservative management versus invasive management of significant traumatic pneumothoraces in the Emergency Department (The CoMiTED Trial): a study protocol for a randomised non-inferiority trial

Nicola M Blythe, Katherine Coates, Jonathan Benger, Ammar Annaw, Jonathan Banks, Clare Clement, Madeleine Clout, Antoinette Edwards, Daisy Gaunt, Rebecca Kandiyali, J Athene Lane, Fiona Lecky, Nick Maskell, Chris Metcalfe, Marie Platt, Sophie Rees, Jodi Taylor, Julian Thompson, Steven Walker, Douglas West and Edward Carlton.

Word count: 4122 (excluding title page, abstract, figures and tables, acknowledgements, contributions and references)

Number of tables: 2

Number of figures: 2 (submitted separately)

Corresponding author: Professor Edward Carlton, Emergency Department, Southmead Hospital, North Bristol NHS Trust, Southmead Road, Bristol, BS10 5NB. Email: ed.carlton@bristol.ac.uk.

Key words: accident and emergency medicine, trauma management, randomised controlled trial, traumatic pneumothorax, conservative management

For peer review only

ABSTRACT

Introduction

Traumatic pneumothoraces are present in 1 in 5 victims of severe trauma. Current guidelines advise chest drain insertion for most traumatic pneumothoraces, although very small pneumothoraces can be managed with observation at the treating clinician's discretion. There remains a large proportion of patients in whom there is clinical uncertainty as to whether an immediate chest drain is required, with no robust evidence to inform practice. Chest drains carry a high risk of complications such as bleeding and infection. The default to invasive treatment may be causing potentially avoidable pain, distress and complications. We are evaluating the clinical and cost-effectiveness of an initial conservative approach to the management of patients with traumatic pneumothoraces.

Methods and analysis

The CoMiTED trial is a multicentre, pragmatic parallel group, individually randomised controlled non-inferiority trial to establish whether initial conservative management of significant traumatic pneumothoraces is non-inferior to invasive management in terms of subsequent emergency pleural interventions, complications, pain, breathlessness, and quality of life. We aim to recruit 750 patients from at least 40 UK NHS hospitals. Patients allocated to the control (invasive management) group will have a chest drain inserted in the emergency department. For those in the intervention (initial conservative management) group, the treating clinician will be advised to manage the participant without chest drain insertion and undertake observation. The primary outcome is a binary measure of the need for one or more subsequent emergency pleural interventions within 30 days of randomisation. Secondary outcomes include complications, cost-effectiveness, patient-reported quality of life and patient and clinician views of the two treatment options; participants are followed up for 6 months.

Ethics and dissemination

This trial received approval from Wales Research Ethics Committee 4 (reference: 22/WA/0118) and the Health Research Authority. Results will be submitted for publication in a peer-reviewed journal.

Trial registration number ISRCTN35574247.

Strengths and Limitations of this study

- This is a pragmatic trial; once the initial decision has been made and patients have been allocated a treatment arm, all subsequent care and interventions are at the discretion of treating clinical teams.
- Patients will be recruited from the whole of the trauma spectrum to ensure results can be generalisable across the diverse trauma population.
- The trial involves economic evaluation to determine the clinical and cost effectiveness of initial conservative management versus invasive management of traumatic pneumothoraces.
- The trial has an integrated qualitative study in order to assess the acceptability of initial conservative management to patients and clinicians.
- Blinding to treatment allocation is not possible for clinicians or participants; only clinicians adjudicating primary outcome and researchers evaluating outcomes for the analyses will be blinded to treatment group.

INTRODUCTION

Injury is a leading cause of death among adults aged <45 years [1]. Traumatic pneumothoraces are present in 1 in 5 victims of severe trauma [2, 3]. We estimate from prior observational and survey work [4, 5] that around half of patients admitted to hospital with traumatic pneumothoraces will be treated with the insertion of a chest drain. Current guidelines advise chest drain insertion for most traumatic pneumothoraces, although very small pneumothoraces can be managed with observation at the treating clinician's discretion [6, 7]. For some patients with very large pneumothoraces, chest drain placement can reduce the risk of cardiorespiratory compromise [8]. However, there remains a large proportion of patients in whom there is clinical uncertainty as to whether an immediate chest drain is required [4]. Chest drains carry a high-risk of complications, such as bleeding and infection, in 15-30% of patients [9]. There is a lack of robust evidence to inform practice, and the default to invasive treatment may cause potentially avoidable patient harm.

In an analysis of >600 patients with traumatic pneumothoraces from 2012 to 2016, obtained from Trauma Audit & Research Network (TARN) data, 90% of patients treated without a chest drain did not require subsequent intervention [5], suggesting a potential role for conservative management. However, in this analysis, 50% of patients were initially treated with a chest drain and there was considerable clinical variation in those selected for this invasive procedure. In a 2020 international survey of 222 emergency physicians [4], utilising clinical vignettes of larger traumatic pneumothoraces, over 60% of clinicians would elect to insert a chest drain in the Emergency Department (ED), even without clinical compromise. Therefore, based on the observational studies and lack of robust data, we designed a randomised controlled trial (RCT) to assess the clinical and cost-effectiveness of an initial conservative approach to the management of patients with traumatic pneumothoraces. If we demonstrate that this approach achieves similar clinical outcomes, it will reduce the use of a painful, invasive and potentially harmful management strategy.

Prior to the start of the trial, we searched Medline for systematic reviews, and Medline, Embase, Cochrane Central, ClincalTrials.gov and the World Health Organisation (WHO) trials registry for trials

For peer review only - http://bmjopen.bmj.com/site/about/guidelines.xhtml

published. One systematic review from 2010 evaluated three small (total n=101) RCTs examining the safety of conservative management in small traumatic pneumothoraces [8]. This review suggested that conservative management may be at least as safe and effective as chest drain insertion. A further multicentre RCT of small pneumothoraces in severely injured patients in Canada concluded in 2021 [10]. These patients (142 in total) were all receiving positive pressure ventilation and current guidelines suggest chest drain insertion in all patients undergoing ventilation [2, 4]. The results showed no difference in mortality or intensive care unit (ICU) or hospital length of stay between patients who were conservatively managed and those who had chest drains inserted. The authors concluded that small traumatic pneumothoraces may be safely observed in patients undergoing ventilation and that the complications of chest drains remain unacceptably high. By including only patients undergoing ventilation (which is around 30% of the traumatic pneumothorax population in the UK [5]), the Canadian study did not fully address conservative management in the broader trauma population, as we are in this trial.

Aims and objectives

The Conservative Management in Traumatic Pneumothoraces in the Emergency Department (CoMiTED) trial will test whether initial conservative management of significant traumatic pneumothoraces is non-inferior to invasive management in terms of subsequent emergency pleural interventions, complications, pain, breathlessness, and quality of life.

Specific objectives are:

a) To establish if initial conservative management is non-inferior to invasive management regarding subsequent emergency pleural intervention over 30 days (or until death if sooner).

b) To determine whether conservative management improves health-related quality of life and other patient reported outcomes.

c) To determine the clinical and cost effectiveness of initial conservative management versus invasive management of traumatic pneumothoraces by measuring resource use, mortality and costs over the six months following injury.

d) To assess acceptability of initial conservative management to patients and clinicians.

METHODS AND ANALYSIS

Trial design

The CoMiTED trial is a pragmatic multicentre, parallel group, individually randomised controlled non-inferiority trial with an economic evaluation and integrated qualitative study.

Setting

The trial will recruit patients from approximately 40 NHS Major Trauma Centres and Trauma Units across the UK.

For peer review only - http://bmjopen.bmj.com/site/about/guidelines.xhtml

Trial population

Inclusion and exclusion are detailed in Table 1.

Inclusion Criteria	Exclusion Criteria
Presenting with traumatic pneumothorax/pneumothoraces	Treating clinician(s) believes injuries are incompatible with life
(Believed to be) 16 years and over	Patient in respiratory arrest
Treating clinician(s) believes either a chest drain or conservative management is a suitable initial treatment option	Haemothorax (associated with pneumothorax) requiring a chest drain in the opinion of the treating clinician(s)*
	Clinical or imaging evidence of tension pneumothorax in either lung at the point of randomisation
	Prisoner

Table 1. Table illustrating inclusion and exclusion criteria. Special Circumstances: In patients presenting with bilateral chest injury, if one lung of the patient qualifies, the patient can be enrolled, providing no exclusion criteria are met. Treatment of the eligible side follows the randomisation assignment, with the other side treated according to usual practice. If both sides qualify, both sides receive treatment according to the randomisation assignment. Patients who have received pre-hospital thoracostomies may still be enrolled, provided they fulfil the eligibility criteria. Where a participant who has received a prehospital thoracostomy is randomised to conservative management, local practice should be followed.

**Patients with an associated haemothorax are excluded due to this being a predictor of failure of conservative management [5].*

Primary outcome

The primary outcome is a binary measure of the need for one or more subsequent emergency pleural interventions in the eligible lung(s), from the point of randomisation up to 30 days. This excludes chest drain insertion in the ED for those allocated to the chest drain (control) group.

Reasons for subsequent emergency chest drain insertion may include (but are not limited to): clinically significant symptoms persisting despite adequate analgesia; chest pain or breathlessness preventing activity; a patient is unwilling to continue with conservative treatment; the patient's condition becomes physiologically unstable presumed secondary to pneumothorax; repeat chest radiograph shows an enlarging pneumothorax with physiological instability. Reasons for subsequent emergency pleural intervention are determined by local practice and recorded but are not controlled.

Whether a subsequent pleural intervention is deemed to be an emergency is adjudicated by a panel made up of independent expert clinicians from relevant specialties. The clinicians are blinded to allocation and presented with clinical and imaging vignettes of what happened to each participant and subsequently asked to determine whether, in their opinion, any subsequent pleural intervention that occurred within 30 days of randomisation was required due to an emergency. Consensus agreement is obtained by two members of the panel.

For peer review only - http://bmjopen.bmj.com/site/about/guidelines.xhtml

Secondary outcomes

The secondary outcomes will capture any differences between the allocated groups in terms of reduced pain, complications and improved health-related quality of life in the short to medium term, as well as inform a formal cost-effectiveness analysis.

Secondary outcomes are as follows: (i) All pleural interventions (including chest drain insertion in the ED) up to 30 days, (ii) All complications of pleural intervention up to 30 days, (iii) Total days of pleural drainage up to 30 days, (iv) Patient-reported pain [11], function and breathlessness [12] at baseline, 30 days, 3 and 6 months, (v) Quality of life [13, 14] at baseline, 30 days, 3 and 6 months, (vi) Total length of stay (hospital, critical care (including HDU) admission and readmission) up to 30 days, (vii) Adjudicated mortality at 30 days (pneumothorax or chest injury related), (viii) All-cause mortality at 6 months, (iv) Cost per quality-adjusted life year (QALY) at 6 months, (x) Patient views and experiences of conservative management/chest drain and (xi) Clinician views of conservative management/chest drain. For this trial, baseline patient reported outcome measures (PROMs) can be collected from as soon as feasible following randomisation and after treatment delivery (where appropriate) up to 7 days post-randomisation.

Sample size

We aim to recruit 750 participants and conduct approximately 25 patient interviews for the integrated qualitative study over 37 months (August 2022 – September 2025).

Observational data suggests that 10% of our trial population will require emergency pleural intervention following conservative management [5]. Our group recently identified a reintervention rate of 10% following initial chest drain insertion in a single UK site [5] and therefore anticipate the incidence of the primary outcome in the control group to be at least 10%.

Our patient and public involvement (PPI) contributors unanimously support the potential advantages of initial conservative management, such as avoiding an invasive procedure, improved mobilisation after injury, and reduced longer term pain. However, they also recognise the need to balance these benefits against the risk of avoidable harm. When asked, our PPI group felt that an increase of 5-10% in subsequent emergency pleural intervention would be acceptable compared to usual care, given the anticipated reduction in the overall number of chest drains in the intervention group. These views have been used to select a non-inferiority margin of 7.5%. We will conclude that the trial population can be safely managed conservatively if the incidence of subsequent emergency pleural intervention is no more than 7.5% higher in the intervention group than in the control group. If the incidence of the primary outcome is 10% in both groups, a sample size of 674 (337 in each group) will allow non-inferiority to be concluded with 90% power, when comparing a one-sided 97.5% confidence interval, for the absolute difference in primary outcome incidence, to a non-inferiority margin of 7.5%. Allowing 10% loss to follow-up increases the total sample size to 750.

Patient approach, recruitment and randomisation

Following eligibility assessment, eligible patients undergo a capacity assessment. If the patient has capacity, they are approached in the ED for their consent to take part. Where patients are judged to be unable to provide informed consent for themselves, then patients can be automatically enrolled under the waiver of consent (in countries where the waiver of consent is permitted for non-CTIMP trials). If patients regain capacity within 7 days post-randomisation, they are approached and asked whether they wish to provide consent to continue in the trial. If patients do not regain capacity

For peer review only - http://bmjopen.bmj.com/site/about/guidelines.xhtml

within 7 days of randomisation, a member of the research team seeks advice from a Personal Consultee or, if unavailable, a Nominated Consultee. The participant pathway is shown in Figure 1.

Patients are randomised in the ED immediately after traumatic pneumothorax has been diagnosed and consent provided / waiver of consent applied. Participants are allocated in a 1:1 ratio to either "chest drain insertion in the ED (control group)" or "initial conservative management (intervention group)" using a secure web-based randomisation system (Sealed Envelope, https://www.sealedenvelope.com/). Randomised allocation is minimised by three factors: 'trial site', 'currently ventilated' and 'penetrating injury'.

Trial intervention

In the intervention (initial conservative management) group, the treating clinician is advised to manage the participant without chest drain insertion and undertake observation and admission to a hospital ward or ICU. Given the pragmatic nature of this trial, all subsequent interventions and further imaging to evaluate pneumothorax resolution after the point of randomisation, is at the discretion of the treating clinical teams.

In the control group (chest drain insertion in the ED), the treating clinician is advised to insert a chest drain. Specific details of the procedure (including anaesthesia and insertion technique) is at the discretion of treating clinicians but will be recorded for trial purposes.

Data collection

Trial data is collected at baseline, 30 days, 3 months and 6 months and recorded by participating site team members onto case report forms (CRFs) and participant-completed questionnaires. Table 2 depicts the key assessments/outcome measures and participant-related procedures scheduled at various trial timepoints. These are entered into a REDCap database [15] for data cleaning and analysis. Access to the database is via a secure password-protected web interface.

**Data collection timepoint (→)**	**In the Emergency Department (ED)**		**Post-Randomisation Follow Ups**		
**Data capture / key trial procedure (↓)**	**Recruitment (Day 0)**	**Post-Recruitment (Baseline)**	**30 days**	**3 months**	**6 months**
Screening, Consent & Randomisation	X				
Case Report Form including safety reporting (CRF)	X	X	X	X	X
Sociodemographic Details		X			
Injury Details		X			
Injury Severity Score		X			
Comorbidities		X			

For peer review only - http://bmjopen.bmj.com/site/about/guidelines.xhtml

National Early Warning Score (routinely collected)		X			
PROMS; Pain (Brief Pain Inventory)		X	X	X	X
PROMS; Function (Brief Pain Inventory)		X	X	X	X
PROMS; Breathlessness (MRC dyspnoea scale)		X	X	X	X
PROMS; Quality of life (EQ-5D-5L)		X	X	X	X
PROMS; Impact Events Scale (IES-R)			X	X	X
Patient completed resource use questionnaire				X	X
Pleural interventions			X		
Complications			X		
Days of pleural drainage			X		
Length of stay (hospital and critical care (including HDU) admission, and readmission)			X		
Details of re-attendances to A&E or unplanned re-admissions within 30 days			X		
Mortality			X		X
Qualitative interviews			X		X

Table 2. Schedule of essential data capture and participant-related procedures.

For peer review only

Statistical analysis

Data obtained will be analysed according to the intention to treat principle, such that each participant's data will contribute to the findings for the group they were allocated to, irrespective of any subsequent diagnostic information or the treatment actually received. Reporting of the trial methodology and results will be according to the CONSORT guidelines [16]. The analysis will be pre-specified in detail in a statistical analysis plan, which will be made publicly available prior to the trial team having access to the data. The findings for the primary outcome measure will be presented as an absolute difference in incidence between conservative management and control groups, with the limit of the one-sided 97.5% confidence interval, compared to the non-inferiority bound of an absolute difference of a 7.5% higher incidence of the primary outcome in the conservative

For peer review only - http://bmjopen.bmj.com/site/about/guidelines.xhtml

management group. The primary analysis will be based on the observed data, but the potential impact of any missing primary outcome measures on the trial conclusions will be investigated in sensitivity analyses. If non-inferiority is demonstrated, evidence from the risk difference, two-sided 95% confidence interval, and p-value, will be presented to allow inference about the superiority of conservative management compared to usual care.

Health economic analysis

A cost utility analysis with a maximal time horizon of 6 months (corresponding to the period of maximal follow-up for patient-reported pain, dyspnoea and mortality) will be undertaken, since this is the time period that clinicians and patient advisors advise us is long enough to capture all relevant effects. However, it is possible that we will see convergence of costs and outcomes within 30 days (which corresponds to the primary outcome), and, to explore this, we will report cost-effectiveness at both 30 days and 6 months.

The QALY will be derived by applying the cross-walk algorithm to the EQ-5D-5L [13] and combining information on survival.

Resource use data is being collected on all NHS and personal social services care resources for trial participants up to 6 months. A patient-reported resource use questionnaire (note that the patient questionnaire will incorporate ModRUM [17], with the addition of some trial-specific questions) at 3 and 6 months will provide additional data on primary and community care resource after discharge.

Utilising medical notes from patients coded for chest drain insertion at one site, a set of assumptions have been established detailing staff, equipment, analgesia and imaging use relating to chest drain insertion and other high-cost pleural interventions in different settings and these will be reviewed by clinicians at participating organisations for accuracy. The aim of this is to enhance our understanding of the trauma pathway and to inform and validate our costing approach.

Qualitative analysis

The qualitative research aims to provide a comprehensive and in-depth understanding of the acceptability of initial conservative management versus chest drain to patients and clinicians by conducting interviews using topic guides; these guides are shown in Supplementary File 1. Patients and consultees will be approached at least eight weeks after randomisation. Interviews will explore patient and consultee views and experiences of conservative management or chest drain insertion in the short to medium term, including impact on their daily life, positive and negative aspects of the treatment, pain, post-procedure recovery, subsequent treatments and return to activities. To enhance the understanding of clinician acceptability of initial conservative treatment and its implementation in wider practice, we will also interview clinicians involved in the trial patient pathway. Interviews will explore views of initial conservative management/chest drain, potential hidden benefits of initial conservative management, barriers to and facilitators for introducing initial conservative management into practice and what influences decision-making concerning traumatic pneumothorax management.

Maximum variation/purposive sampling will be used when possible, with the aim of achieving diversity in terms of participant characteristics. Anonymised transcripts will be analysed using reflexive thematic analysis [18]. Transcripts will be coded for key categories and concepts, using deductive coding (based on the research aims and Theoretical Framework of Acceptability) [19] and inductive coding (developing new codes based on issues emerging from the data) with the aid of

For peer review only - http://bmjopen.bmj.com/site/about/guidelines.xhtml

NVivo software. Findings will be considered in relation to quantitative results and provide enhanced understanding of chest injury management in the emergency context.

Safety

Participant safety will be monitored by the Trial Management Group (TMG), Sponsor and oversight committees (Trial Steering Committee and Data Monitoring Committee). The protocol contains a list of events that can be expected in this patient population. If an expected serious adverse event (SAE) is prolonged or more serious than expected, this will be reported as an unexpected SAE.

All SAEs, expected non-serious adverse events (AEs) and non-serious AEs caused by pleural interventions (which occur up to 30 days post-randomisation for the latter) will be recorded in CRFs and monitored. SAEs that are both related to the trial (i.e. resulted from conservative management or administration of a research procedure) and unexpected (i.e. not listed in the protocol as expected) are suspected unexpected serious adverse reactions (SUSARs) and will be subject to reporting to the Sponsor and Research Ethics Committee (REC). We do not expect any AEs or SAEs related to conservative management (above those expected of the control arm, i.e. standard care).

Patient and Public Involvement

A PPI group made up of PPI co-applicants/members, and supplemented through networking and outreach work, meet as needed throughout the duration of the trial to ensure an iterative and responsive PPI strategy. Our PPI group have fed into all aspects of trial design, provided feedback on trial documents and have been involved in maximising retention of participants. A group of knife crime and violence reduction professionals from a boxing group in Bristol, Empire Fighting Chance, meet separately to address this important element of the trial.

Trial management and oversight

The Chief Investigators take overall responsibility for managing the trial. Bristol Trials Centre, a UK Clinical Research Collaboration-registered trials unit, is responsible for the preparation of trial documents, site initiation visits and training, day-to-day running of the trial and monitoring of centres. The TMG oversees the trial and meets bimonthly to review progress. The trial steering committee meets biannually to review conduct and progress and the data monitoring committee at least annually to review data completion and safety. The trial Sponsor is North Bristol NHS Trust, who oversees the trial and has ultimate responsibility for any decision about its continuation.

Changes to trial protocol

Since the first trial protocol was approved by the Research and Ethics Committee (V2.0, dated 11 May 2022), there have been three amendments to the protocol (current version is version 5.0, dated 08 June 2023). The first amendment (protocol version 3.0, 07 July 2022) clarified the eligibility criteria where bilateral pneumothoraces are present. The second amendment (protocol version 4.0, 15 December 2022), amended the key inclusion criterion from '*in whom the treating clinician(s) are uncertain if a chest drain is required*' to '*in whom the treating clinician(s) believes either a chest drain or conservative management is a suitable initial treatment option*', based on feedback from participating organisations. The third amendment, (protocol version 5.0, 08 June 2023), allowed the recruitment of patients at NHS organisations in Scotland, via informed consent only, due to differences in legalities for patients judged not to have capacity in Scotland.

For peer review only - http://bmjopen.bmj.com/site/about/guidelines.xhtml

DISCUSSION

This trial investigating initial conservative management of traumatic pneumothoraces is a pragmatic multicentre RCT aiming to establish whether this approach is non-inferior to chest drain insertion in terms of clinical and cost-effectiveness. Should an initial conservative approach prove non-inferior to invasive management, this is likely to lead to widespread changes in practice and reduce avoidable harm from chest drain insertion.

We recognise that this trial is both methodologically complex and will be a challenge to deliver in an emergency setting. The following aspects have been considered in order to ensure the trial can be successfully delivered and answer the aims and objectives.

Clinical Equipoise

Equipoise is the key to our third inclusion criterion which relates to whether the treating clinician(s) would feel comfortable treating a patient's traumatic pneumothorax with a chest drain or conservative management. The subjective nature of this inclusion criterion has been our most significant challenge to date. During the initial stages of recruitment, this inclusion criterion read '*in whom the treating clinician(s) are uncertain if a chest drain is required*'. During the early stages of recruitment, both the trial team and the qualitative research team received feedback from site teams that clinicians may have been perceiving this as questioning their confidence in their clinical decision-making, rather than the intended 'research uncertainty', and that eligible patients may be being excluded due to this. An amendment was implemented to change this key inclusion criterion to '*in whom the treating clinician(s) believes either a chest drain or conservative management is a suitable initial treatment option*'. The trial team have emphasised when training site teams that a patient should be considered if the treating clinician acknowledges that the patient could be treated differently if seen by a colleague, or if they presented at a different NHS hospital. During training, case studies (anonymised radiology images) are shared with sites to illustrate the variation in the sizes of traumatic pneumothoraces within the trial, including mention of factors which affected decision-making (e.g. presence of surgical emphysema, ventilation status, body mass index).

In addition, variation in embedded practice within the specialty groups involved in decision-making has been noticeable throughout the duration of the trial. Generally speaking, emergency clinicians seem more comfortable with treating small to moderate traumatic pneumothoraces conservatively, whereas there has been some reluctance from the surgical community, with a preference for chest drain insertion often observed. This may be due to concerns about the potential increased need for chest drain insertion on hospital wards and the resource available to do this. Site teams have been reassured that the number of patients recruited at each site will be relatively small, that only half of the patients will be allocated to the conservative management arm and, in addition, the need for subsequent intervention is likely to be low at ~1 in 10, based on previous observational data [5].

Recruitment of multiply-injured patients

We anticipated that 40% of participants recruited would be intubated and ventilated, based on TARN data. However, in June 2023, analysis showed that only 8% of patients recruited were intubated and ventilated at the time of randomisation. This may have been due to a preference for invasive management in positively pressure ventilated patients, despite prior evidence demonstrating that ventilation does not predict failure of conservative management [5,10]. The TMG were concerned that this may affect the generalisability of the trial's results. The trial team

For peer review only - http://bmjopen.bmj.com/site/about/guidelines.xhtml

have been engaging the critical care community via infographics and webinars and have since seen an increase in the proportion of intubated and ventilated patients included to 15%.

Trial information provision

Due to the heterogenous target population, we have trial information material available in a variety of formats to facilitate maximal participation in eligible patients. We created a patient information video (https://comited.blogs.bristol.ac.uk/information-for-patients/) which was aimed towards younger people. Patients are able to provide consent to participate after watching the video, with a detailed patient information sheet also provided for further reading. Individuals from the charity Empire Fighting Chance made a valuable contribution towards creation of the patient information video, providing feedback and suggestions of ways to ensure the video was relevant to the target group. The video has received positive feedback from participating site research teams and has enabled at least one patient who was unable to read to participate in the trial.

There are two pathways via which to enrol a patient into the trial: obtaining informed consent from those with capacity or recruiting those lacking capacity (temporarily or permanently) under the emergency waiver of consent [20]. In some cases, patients may initially seem alert (e.g. be standing, walking or talking), and this may be mistaken for capacity to make an informed decision about participation in research, especially in those who are under the influence of alcohol or drugs (recreational or medication) or in extreme pain. We have found that, in some situations, such patients do not recall the 'informed consent' discussion when spoken to at a later date so we encourage site teams to keep this in mind and to enrol patients under the waiver of consent if they feel this is appropriate.

The CoMiTED research team, alongside collaborators, created a short video for those who were temporarily lacking capacity at the time they were admitted to the ED and enrolled under the waiver of consent (https://comited.blogs.bristol.ac.uk/for-patients-who-have-recovered-capacity/). The video explains that they were too unwell to be asked about taking part at the time a decision about their treatment needed to be made, therefore doctors decided that it was safe for the patient to take part. The video explains that, now that the patient is well enough, they are being approached with details of the trial and being asked if they wish to provide consent for continued participation. The video is not specific to the CoMiTED trial and is available for use in all emergency care trials utilising the waiver of consent.

Conclusion

The CoMiTED trial is a multicentre pragmatic RCT which has been designed to generate new evidence around the management of patients with traumatic pneumothoraces and has the potential to lead to significant changes in clinical practice.

Trial status

Recruitment to the trial began in August 2022, with an internal pilot to test feasibility. The trial is currently recruiting at 41 UK organisations (20 major trauma centres and 21 trauma units, distribution is shown in Figure 2); and, as of 10/04/2024, 235 participants have been recruited across 35 sites.

For peer review only - http://bmjopen.bmj.com/site/about/guidelines.xhtml

Ethics and dissemination

This trial was given a favourable opinion by Wales Research Ethics Committee 4 (reference: 22/WA/0118) and received approval from the Health Research Authority.

Trial participants are kept informed of trial progress via newsletters. A trial-specific X (previously Twitter) account, @CoMiTEDTrial, is used to promote the trial, provide updates and will disseminate findings. We aim to publish our primary, peer-reviewed manuscript in a high impact medical journal and present our findings at multiple conferences. We will communicate our findings to the British Thoracic Society, National Institute for Health and Care Excellence and NHS England to incorporate the work into relevant national guidelines. The dataset will be published in the publicly available University of Bristol Research Data repository https://www.bristol.ac.uk/staff/researchers/data/accessing-research-data/.

Acknowledgments

The authors thank all of the research and clinical team members at participating sites, members of the independent Trial Steering Committee, Data Monitoring Committee and adjudication panel, members of the patient and public involvement group and all of the participants who have taken part in this research so far. The authors would also like to thank Kate Beckett, Holly Mckeon and Amanda Lewis for their contributions.

Contributors

EC and JB are the Co-Chief Investigators, identified the funding opportunity and designed the trial, alongside co-applicants. NB set-up and manages the day-to-day running of the trial and assembled the manuscript from the trial protocol. KC and MP manage the day-to-day running of the trial. CM supervises the statistical analysis, and will write the statistical analysis plan. DG provides progress reports to the Trial Management Group and the independent oversight committees, and will complete the statistical analysis. SR leads the qualitative research, alongside JBa and, previously, CC. RK leads the health economics research, alongside AA. JT, AL & MC provide senior trial management oversight and advice. AE, FL, NM, SW, JTh and DW provide advice as part of the Trial Management Group. All authors were involved in preparation of the trial protocol and have read and approved the final manuscript.

Funding

The project is funded by the National Institute for Health and Care Research (NIHR) Health Technology Assessment Programme (HTA) (ref: NIHR132889). The views expressed are those of the authors and not necessarily those of the NIHR or the Department of Health and Social Care. This trial has been designed and delivered in collaboration with Bristol Trials Centre, a UK Clinical Research Collaboration-registered clinical trials unit, which is in receipt of NIHR clinical trials unit support funding.

Competing interests

The authors declare that they have no competing interests.

For peer review only

References

1. Moran C, Lecky F, Bouamra O, et al. Changing the System – Major Trauma Patients and Their Outcomes in the NHS (England) 2008–17. Lancet Eclinical Medicine. 2018;2:13-21.
2. Di Bartolomeo S, Sanson G, Nardi G, et al. A population-based study on pneumothorax in severely traumatized patients. J Trauma. 2001;51(4):677-82.
3. Kshettry VR, Bolman RM, 3rd. Chest trauma. Assessment, diagnosis, and management. Clin Chest Med. 1994;15(1):137-46.
4. Avery P, Watkins M, Benger J, et al. Current management of moderate to severe traumatic pneumothoraces: a survey of emergency clinicians. Emerg Med J. 2021.
5. Walker SP, Barratt SL, Thompson J, et al. Conservative Management in Traumatic Pneumothoraces: An Observational Study. Chest. 2018;153(4):946-53.
6. Surgeons Aco. Advanced Trauma Life Support® Student Course Manual. 10th Edition ed:Chicago: American College of Surgeons; 2018.
7. Thies KMG, P. European Trauma Course 4th edition. 2016.
8. Yadav K, Jalili M, Zehtabchi S. Management of traumatic occult pneumothorax. Resuscitation. 2010;81(9):1063-8.
9. Menger R, Telford G, Kim P, et al. Complications following thoracic trauma managed with tube thoracostomy. Injury. 2012;43(1):46-50.
10. Clements TW, Sirois M, Parry N, et al. OPTICC: A multicentre trial of Occult Pneumothoraces subjected to mechanical ventilation: The final report. American journal of surgery. 2021;221(6).
11. Tan G, Jensen MP, Thornby JI, at al. Validation of the Brief Pain Inventory for chronic nonmalignant pain. J Pain. 2004;5(2):133-7.
12. Stenton C. The MRC breathlessness scale. Occupational Medicine. 2008;58(3):226-7.
13. EuroQol G. EuroQol--a new facility for the measurement of health-related quality of life. Health Policy. 1990;16(3):199-208.
14. Weiss DS, Marmar C, Wilson JP, et al. Assessing psychological trauma and PTSD. 1997.
15. Harris PA, Taylor R, Thielke R, et al. Research electronic data capture (REDCap)–a metadata-driven methodology and workflow process for providing translational research informatics support. J Biomed Inform. 2009;42(2):377–81.
16. CONSORT Group. Consort transparent reporting of trials, 2010. Available: http://www. consort- statement.org/
17. Garfield K, Husbands S, Thorn JC, et al. Development of a brief, generic, modular resource-use measure (ModRUM): cognitive interviews with patients. BMC Health Services Research. 2021;21(1):1-12.
18. Braun V, Clarke V. Reflecting on reflexive thematic analysis. Qualitative Research in Sport, Exercise and Health. 2019;11(4):589-97.
19. Sekhon M, Cartwright M, Francis JJ. Acceptability of healthcare interventions: an overview of reviews and development of a theoretical framework. BMC Health Serv Res. 2017;17(1):88.
20. Health Research Authority (HRA). Principles of consent: Emergency Research (England and Wales) [19 November 2021]. Available from: http://www.hradecisiontools.org.uk/consent/principles-emergency-EnglandandWales.html.

For peer review only - http://bmjopen.bmj.com/site/about/guidelines.xhtml

Figure legends (figures submitted separately)

Figure 1. Trial schema illustrating the pathway for CoMiTED participants.

**It should be noted that patients without capacity are not being recruited in Scotland (Pathway B in England, Wales and Northern Ireland only).*

Figure 2. Distribution of participating organisations in the UK. Closed circles indicate major trauma centres and open circles indicate trauma units.

For peer review only

For peer review only - http://bmjopen.bmj.com/site/about/guidelines.xhtml

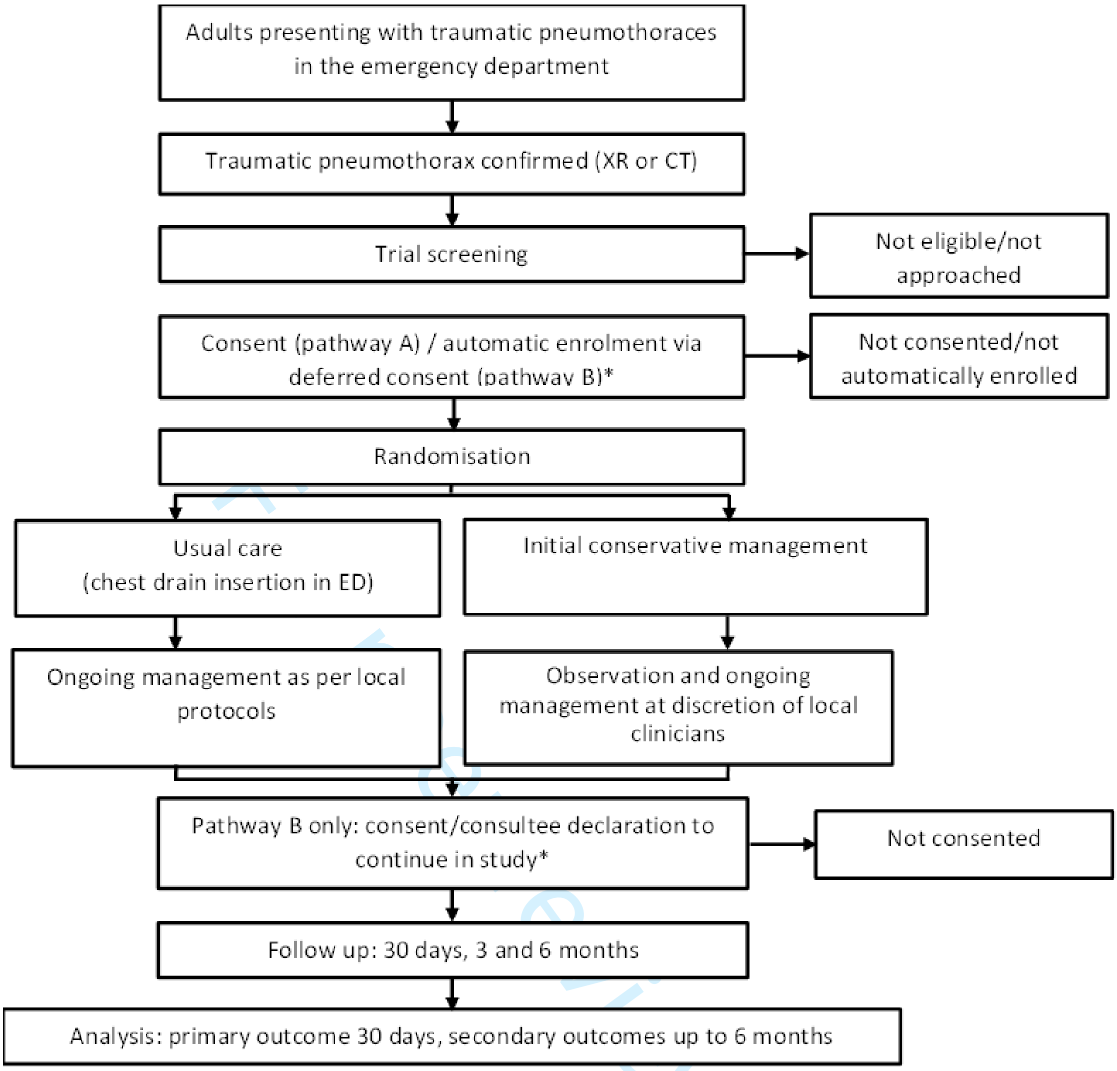

Figure 1. Trial schema illustrating the pathway for CoMiTED participants.

**It should be noted that patients without capacity are not being recruited in Scotland (Pathway B in England, Wales and Northern Ireland only).*

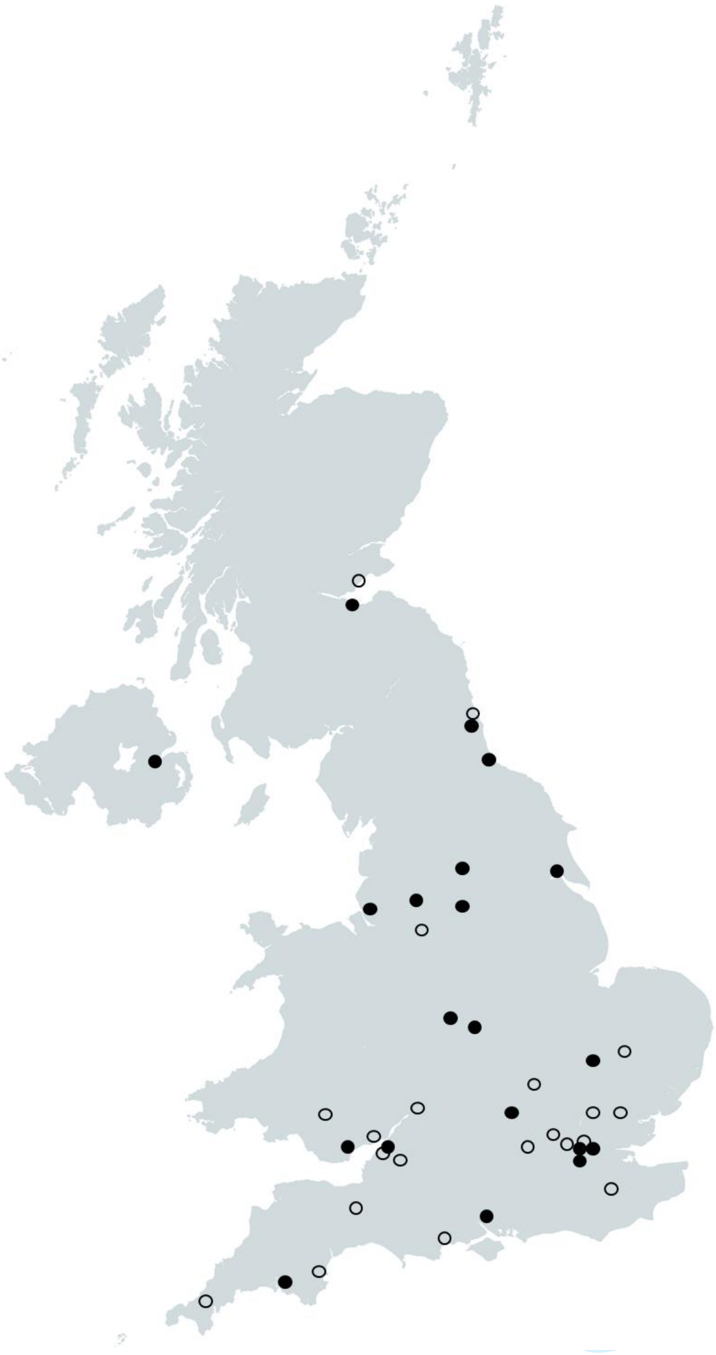

Figure 2. Distribution of participating organisations in the UK. Closed circles indicate major trauma centres and open circles indicate trauma units.

CoMiTED topic guides

Trial participant/consultee interviews

Background information on participant e.g., age, typical day, [consultees: – relationship to participant/carer responsibilities]

- **Experience leading to participation**
    - Can you tell me about how you/your relative (as appropriate) came to be admitted to the hospital? What happened? What were the injuries? If no memory of admittance – explore from when they can remember.
    - What happened to you/them after you/they were admitted to the hospital?

- **Trial views**
- Can you tell me about when you first heard about the CoMiTED study? Where, when, who?
    - **How was the study explained to you?**
    - Did you see a video about the study? What are your thoughts about the video? Good/bad/helpful?
- What did you think about the study? good idea/bad/any concerns?
- What is your understanding of the CoMiTED study? What are the researchers trying to do and why?
- Did the study make sense to you? probe why, why not – anything worried about?
- Could you tell me your thoughts on being involved in the study? Anything worried about? Anything they like about it?
    - How did feel about randomisation
    - If personal consultee, explore their understanding of their role and what is involved
    - Prompt paperwork/questionnaires
- Why did they decide to take part in the study/become a personal consultee?
- Thoughts on the consent process? Explore different routes
    - if were enrolled via deferred consent, explore their thoughts on this – understanding of/thoughts about?
- Why did you decide to take part (continue to take part if deferred consent)?
- Are you glad you took part in the study? Why, why not? Would you take part in the study again? Would you recommend family/friends to take part in the study? Explore.
- Questionnaires – any feedback, got on ok with them?
- Challenges/what could have improved your experience of taking part?
- Is there anything else you would have liked more information on?
- Are you glad you took part in the study? Why, why not? Would you do it again? Would you recommend family/friends to take part in the study? Explore. Explore whether and why views may have changed over time.

- **Treatment/symptom experiences**
- Explore treatments – chest drain/not when admitted – how long had drain for?
- What happened in the early days following the procedure/treatment, pain management, outcomes?
- Explore what they understand about the treatments (chest drain or not) they received and why they had it at the time of injury. Positive and negative aspects of the intervention, experience of

For peer review only - http://bmjopen.bmj.com/site/about/guidelines.xhtml

CoMiTED

pain and its management, information provided, support available. Any subsequent medical visits/treatments? Experience of discharge.

- What was your experience of recovery following the procedure/treatment (since joining CoMiTED)?
- prompts: what happened after left hospital, earlier months – pain management, outcomes. Satisfaction with discharge? Were they told what to expect? Was this sufficient and met expectations?
- Describe any changes you have experienced in your symptoms up to now?
- prompt – duration, experiences of pain, breathlessness, functioning – changes in symptom experience, new onset symptoms?
- Have your symptoms changed much over the last few months (i.e. 3 vs 6 months)
- What symptoms are you experiencing now?
    - prompts – onset, duration, experiences of pain, breathlessness, functioning
- How do the symptoms bother you?
    - prompts – explore why bother them, what aspects of treatments more/less bothersome,
- How are symptoms affecting your daily life?
    - What impact has injury & treatment had on life
        - prompt – quality of life, personal costs e.g., sleep, social/other activities, emotional wellbeing, work, impact on family, relationships
        - Effect on self-perception, perception of others (e.g. scarring)
    - How have you been managing symptoms?
    - How long do they think the symptoms will last?
- What is their experiences of the treatments?
- Positive and negative aspects of the intervention, experience of pain and its management, information provided, support available. Any subsequent medical visits/treatments?

- **Final thoughts**
    - Thank you so much for your discussion. Do you have any final points that you would like to discuss or that you feel you didn't have the opportunity to talk about?

Health professional interviews

Background information e.g., HCP role, involvement in trial, years' experience,

Topics to be covered

i. Experiences of their participation in the trial

- How heard about the trial/why became involved in the trial
- Do you think there is a need for the CoMiTED study or not? Why?
- Which activities/processes been involved in – describe your experience of these

ii. Views, acceptability and decision-making for initial conservative management

- What is your normal decision-making process around using a chest drain/conservative management for traumatic pneumothoraces?
    - Prompts: What triggers a decision to treat, what informs your decision regarding treatment decisions – chest drain/initial conservative management, what are your typical treatment choices for people admitted to ED for traumatic pneumothoraces?

For peer review only - http://bmjopen.bmj.com/site/about/guidelines.xhtml

- What are your thoughts/perspectives surrounding chest drain?
    - Prompts: perceptions regarding outcomes, appropriateness for which patients, procedure/technical considerations, when would you use it and why? Perceptions regarding symptom resolution, recovery from procedure, patient experiences, impact on quality of life.
- What are your thoughts/perspectives surrounding initial conservative management?
    - Prompts: perceptions regarding outcomes, appropriateness for which patients, procedure/technical considerations, when would you use it and why? Perceptions regarding symptom resolution, recovery from procedure, patient experiences, impact on quality of life.
- Have your perceptions changed surrounding the treatments being evaluated in CoMiTED throughout the study?
    - Prompts: patients may receive different treatments than usually advocated by clinician, have they been surprised by the outcomes, are outcomes as expected, how has the study altered their decision-making considerations, any different thoughts regarding the procedures compared to the start of the study?
- If the trial was to show a benefit to patients between the two different treatment approaches, how do you think this might alter your practice?
    - Prompts: what might you consider differently when deciding between chest drain and initial conservative management, would the findings persuade you to consider different options you may not have had before, what do you think the benefits of using initial conservative management within wider practice may be? Do you think this will be implementable, what might be the difficulties with implementing the trial findings in practice?

iii. Back to the trial

- How would you say CoMiTED has been going at your site? What have been the obstacles to getting going with CoMiTED?
- What has gone well? Any advice for other sites?
- How are decisions about whether the patient is eligible and should be entered into the trial made at your site? Who is involved? Have there been any issues? Overcome?
- How do you think having a multidisciplinary team approach to trauma care affects recruitment to the study? Difficulties? How these can be overcome?
- What are your thoughts on the eligibility criteria? Any issues?
- What might, in your opinion, improve running the trial at your site/other sites? (if not raised at this site, talk about how lack of equipoise has been an issue – any issue here_ How do they think this may be engaged with and tackled in CoMiTED?
- What are your thoughts on randomising patients within CoMiTED?
    - Appropriateness? Concerns? Patients responses?
- Are there instances when you didn't follow the randomisation allocation? Why? Explore reasons
- Views and experiences of the informed consent processes/different pathways including the videos
- Views and experiences of data collection (questionnaires/CRFs)
- Is there anything that we could do to improve about the study?

iv. Concluding interview

- Anything else to add, anything missed, important to capture?
- Thank them. Reassure of confidentiality

For peer review only - http://bmjopen.bmj.com/site/about/guidelines.xhtml

SPIRIT

STANDARD PROTOCOL ITEMS: RECOMMENDATIONS FOR INTERVENTIONAL TRIALS

SPIRIT 2013 Checklist: Recommended items to address in a clinical trial protocol and related documents*

Section/item	ItemNo	Description	Addressed on page number
**Administrative information**			
Title	1	Descriptive title identifying the study design, population, interventions, and, if applicable, trial acronym	1
Trial registration	2a	Trial identifier and registry name. If not yet registered, name of intended registry	2
	2b	All items from the World Health Organization Trial Registration Data Set	N/A
Protocol version	3	Date and version identifier	9
Funding	4	Sources and types of financial, material, and other support	12
Roles and responsibilities	5a	Names, affiliations, and roles of protocol contributors	1, 12
	5b	Name and contact information for the trial sponsor	9
	5c	Role of study sponsor and funders, if any, in study design; collection, management, analysis, and interpretation of data; writing of the report; and the decision to submit the report for publication, including whether they will have ultimate authority over any of these activities	8, 9
	5d	Composition, roles, and responsibilities of the coordinating centre, steering committee, endpoint adjudication committee, data management team, and other individuals or groups overseeing the trial, if applicable (see Item 21a for data monitoring committee)	4, 8, 9

For peer review only

**Introduction**			
Background and rationale	6a	Description of research question and justification for undertaking the trial, including summary of relevant studies (published and unpublished) examining benefits and harms for each intervention	2, 3
	6b	Explanation for choice of comparators	2, 3
Objectives	7	Specific objectives or hypotheses	3
Trial design	8	Description of trial design including type of trial (eg, parallel group, crossover, factorial, single group), allocation ratio, and framework (eg, superiority, equivalence, noninferiority, exploratory)	3
**Methods: Participants, interventions, and outcomes**			
Study setting	9	Description of study settings (eg, community clinic, academic hospital) and list of countries where data will be collected. Reference to where list of study sites can be obtained	3, Figure 2
Eligibility criteria	10	Inclusion and exclusion criteria for participants. If applicable, eligibility criteria for study centres and individuals who will perform the interventions (eg, surgeons, psychotherapists)	Table 1
Interventions	11a	Interventions for each group with sufficient detail to allow replication, including how and when they will be administered	6
	11b	Criteria for discontinuing or modifying allocated interventions for a given trial participant (eg, drug dose change in response to harms, participant request, or improving/worsening disease)	6
	11c	Strategies to improve adherence to intervention protocols, and any procedures for monitoring adherence (eg, drug tablet return, laboratory tests)	6
	11d	Relevant concomitant care and interventions that are permitted or prohibited during the trial	6

For peer review only - http://bmjopen.bmj.com/site/about/guidelines.xhtml

Outcomes	12	Primary, secondary, and other outcomes, including the specific measurement variable (eg, systolic blood pressure), analysis metric (eg, change from baseline, final value, time to event), method of aggregation (eg, median, proportion), and time point for each outcome. Explanation of the clinical relevance of chosen efficacy and harm outcomes is strongly recommended	4, 5
Participant timeline	13	Time schedule of enrolment, interventions (including any run-ins and washouts), assessments, and visits for participants. A schematic diagram is highly recommended (see Figure)	Figure 1
Sample size	14	Estimated number of participants needed to achieve study objectives and how it was determined, including clinical and statistical assumptions supporting any sample size calculations	5
Recruitment	15	Strategies for achieving adequate participant enrolment to reach target sample size	5
**Methods: Assignment of interventions (for controlled trials)**			
Allocation:			
Sequence generation	16a	Method of generating the allocation sequence (eg, computer-generated random numbers), and list of any factors for stratification. To reduce predictability of a random sequence, details of any planned restriction (eg, blocking) should be provided in a separate document that is unavailable to those who enrol participants or assign interventions	8
Allocation concealment mechanism	16b	Mechanism of implementing the allocation sequence (eg, central telephone; sequentially numbered, opaque, sealed envelopes), describing any steps to conceal the sequence until interventions are assigned	6
Implementation	16c	Who will generate the allocation sequence, who will enrol participants, and who will assign participants to interventions	6
Blinding (masking)	17a	Who will be blinded after assignment to interventions (eg, trial participants, care providers, outcome assessors, data analysts), and how	2, 4

For peer review only - http://bmjopen.bmj.com/site/about/guidelines.xhtml

	17b	If blinded, circumstances under which unblinding is permissible, and procedure for revealing a participant's allocated intervention during the trial	N/A
**Methods: Data collection, management, and analysis**			
Data collection methods	18a	Plans for assessment and collection of outcome, baseline, and other trial data, including any related processes to promote data quality (eg, duplicate measurements, training of assessors) and a description of study instruments (eg, questionnaires, laboratory tests) along with their reliability and validity, if known. Reference to where data collection forms can be found, if not in the protocol	4, 5, 8, Table 2, Figure 1
	18b	Plans to promote participant retention and complete follow-up, including list of any outcome data to be collected for participants who discontinue or deviate from intervention protocols	6, 7
Data management	19	Plans for data entry, coding, security, and storage, including any related processes to promote data quality (eg, double data entry; range checks for data values). Reference to where details of data management procedures can be found, if not in the protocol	6
Statistical methods	20a	Statistical methods for analysing primary and secondary outcomes. Reference to where other details of the statistical analysis plan can be found, if not in the protocol	7, 8
	20b	Methods for any additional analyses (eg, subgroup and adjusted analyses)	7
	20c	Definition of analysis population relating to protocol non-adherence (eg, as randomised analysis), and any statistical methods to handle missing data (eg, multiple imputation)	7
**Methods: Monitoring**			
Data monitoring	21a	Composition of data monitoring committee (DMC); summary of its role and reporting structure; statement of whether it is independent from the sponsor and competing interests; and reference to where further details about its charter can be found, if not in the protocol. Alternatively, an explanation of why a DMC is not needed	9

	21b	Description of any interim analyses and stopping guidelines, including who will have access to these interim results and make the final decision to terminate the trial	N/A
Harms	22	Plans for collecting, assessing, reporting, and managing solicited and spontaneously reported adverse events and other unintended effects of trial interventions or trial conduct	8, 9
Auditing	23	Frequency and procedures for auditing trial conduct, if any, and whether the process will be independent from investigators and the sponsor	N/A
**Ethics and dissemination**			
Research ethics approval	24	Plans for seeking research ethics committee/institutional review board (REC/IRB) approval	11
Protocol amendments	25	Plans for communicating important protocol modifications (eg, changes to eligibility criteria, outcomes, analyses) to relevant parties (eg, investigators, REC/IRBs, trial participants, trial registries, journals, regulators)	11
Consent or assent	26a	Who will obtain informed consent or assent from potential trial participants or authorised surrogates, and how (see Item 32)	5, 6
	26b	Additional consent provisions for collection and use of participant data and biological specimens in ancillary studies, if applicable	N/A
Confidentiality	27	How personal information about potential and enrolled participants will be collected, shared, and maintained in order to protect confidentiality before, during, and after the trial	6, 8
Declaration of interests	28	Financial and other competing interests for principal investigators for the overall trial and each study site	12
Access to data	29	Statement of who will have access to the final trial dataset, and disclosure of contractual agreements that limit such access for investigators	11

For peer review only

Ancillary and post-trial care	30	Provisions, if any, for ancillary and post-trial care, and for compensation to those who suffer harm from trial participation	N/A
Dissemination policy	31a	Plans for investigators and sponsor to communicate trial results to participants, healthcare professionals, the public, and other relevant groups (eg, via publication, reporting in results databases, or other data sharing arrangements), including any publication restrictions	11
	31b	Authorship eligibility guidelines and any intended use of professional writers	N/A
	31c	Plans, if any, for granting public access to the full protocol, participant-level dataset, and statistical code	11
**Appendices**			
Informed consent materials	32	Model consent form and other related documentation given to participants and authorised surrogates	Upon request comited-trial@bristol.ac.uk
Biological specimens	33	Plans for collection, laboratory evaluation, and storage of biological specimens for genetic or molecular analysis in the current trial and for future use in ancillary studies, if applicable	N/A

*It is strongly recommended that this checklist be read in conjunction with the SPIRIT 2013 Explanation & Elaboration for important clarification on the items. Amendments to the protocol should be tracked and dated. The SPIRIT checklist is copyrighted by the SPIRIT Group under the Creative Commons "Attribution-NonCommercial-NoDerivs 3.0 Unported" license.

For peer review only
