# Supplementary material for: Conservative management versus invasive management of significant traumatic pneumothoraces in the emergency department (the CoMiTED trial): a study protocol for a randomised non-inferiority trial
Source: BMJ Open. 2024 Jun 17;14(6):e087464. doi: 10.1136/bmjopen-2024-087464 (PMC11191772; doi:10.1136/bmjopen-2024-087464)
Supplement: Reviewer comments [file bmjopen-2024-087464.reviewer_comments.pdf]

PEER REVIEW HISTORY

BMJ Open publishes all reviews undertaken for accepted manuscripts. Reviewers are asked to complete a checklist review form (**http://bmjopen.bmj.com/site/about/resources/checklist.pdf**) and are provided with free text boxes to elaborate on their assessment. These free text comments are reproduced below.

ARTICLE DETAILS

**TITLE (PROVISIONAL)**	Conservative management versus invasive management of significant traumatic pneumothoraces in the Emergency Department (The CoMiTED Trial): a study protocol for a randomised non-inferiority trial
**AUTHORS**	Blythe, Nicola; Coates, Katherine; Benger, Jonathan; Annaw, Ammar; Banks, Jonathan; Clement, Clare; Clout, Madeleine; Edwards, Antoinette; Gaunt, Daisy; Kandiyali, Rebecca; Lane, Athene; Lecky, Fiona; Maskell, Nick; Metcalfe, Chris; Platt, Marie; Rees, Sophie; Taylor, Jodi; Thompson, Julian; Walker, Steven; West, Douglas; Carlton, Edward

This article was not externally reviewed at BMJ Open. Protocols that have been independently assessed prior to submission to BMJ Open are usually fast-tracked to publication on the grounds that further substantial changes will not be possible. This independent assessment will usually be external, independent review for both a substantial grant award from a non-commercial or government funder and ethics approval.
